# Efficient Current‐Driven Perpendicular Magnetization Switching Through the Synergy of the Orbital and Spin Hall Effects

**DOI:** 10.1002/advs.76805

**Published:** 2026-07-28

**Authors:** Chuangwen Wu, Jing Zhou, Yibo Fan, Jiali Chen, Rongce Sun, Zonghao Deng, Zengxin Wei, Chuantong Ren, Xingen Zheng, Zhaowei Zhang, Jing Zhang, Jingfeng Li, Jinkui Zhao, Wei Jiang, Haibo Ke, Hao Wu, Weihua Wang

**Affiliations:** ^1^ Dongguan Institute of Materials Science and Technology Chinese Academy of Sciences Dongguan Guangdong China; ^2^ Songshan Lake Materials Laboratory Dongguan Guangdong China; ^3^ School of Physical Sciences and Department of Physics School of Sciences Great Bay University Dongguan China; ^4^ Key Laboratory of Advanced Optoelectronic Quantum Architecture and Measurement (MOE) School of Physics Beijing Institute of Technology Beijing China

**Keywords:** charge‐to‐spin conversion, orbital hall effect, perpendicular magnetization switching, spin–orbit torque

## Abstract

Manipulating perpendicular magnetization via electrical current is a promising approach for developing low‐power and ultrafast spintronic applications. However, the increasing difficulty with improving the efficiency associated with conventional spin–orbit torque mechanisms hinders the development of low‐power spintronic device integration. In this study, we demonstrate highly efficient charge‐to‐spin conversion in NiNb alloys by integrating strong spin Hall effect form nickel (Ni) with strong orbital Hall (OHE) effect from niobium (Nb). By systematically tuning the alloy composition, the relative strengths of the orbital Hall effect and spin–orbit coupling (SOC) can be modulated, enabling controllable and enhanced efficiency of charge‐to‐spin conversion. The spin torque efficiency in NiNb alloys reaches as high as −1.4 to −1.7, with the maximum value observed at the equiatomic composition Ni_50_Nb_50_. Our results demonstrate that the integration of elements with intrinsically strong OHE and SOC is a promising way toward efficiency and controllable charge‐to‐spin conversion. This approach provides a generalizable routine for the material design of spin current sources for the next‐generation spintronic devices.

## Graphical abstract

NiNb alloys enable efficient charge‐to‐spin conversion by combining the spin Hall effect of nickel with the orbital Hall effect of niobium. Composition tuning yields spin‐torque efficiencies of −1.4 to −1.7 and a non‐monotonic efficiency‐resistivity relationship, revealing orbital‐spin synergy beyond conventional spin Hall materials and offering a materials‐design route for low‐power spin‐current sources in next‐generation spintronic devices and scalable device integration.

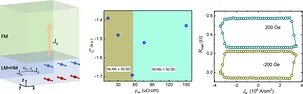

## 1 Introduction

Manipulating magnetization through spin torque is a critical approach in spintronics, enabling high‐density and energy‐efficient magnetic storage and logic operations, especially in the perpendicular magnetic anisotropy (PMA) systems [1, 2, 3, 4, 5]. Subsequently, the efficiency of charge‐to‐spin conversion plays a pivotal role in determining device performance [6, 7, 8, 9, 10]. As illustrated in Figure 1a, in the spin–orbit torque (SOT) systems, the heavy metals (HMs), such as Pt, Ta, and W, work as strong spin–orbit coupling (SOC) materials and convert charge current into spin current through the spin Hall effect (SHE) or interfacial Rashba effect [9, 10, 11, 12, 13, 14]. The resulting spin current is injected into the adjacent ferromagnetic (FM) layer, where it transfers its spin angular momentum to the magnetic moments in the form of a damping‐like torque and a field‐like torque. Identifying HMs that generate stronger spin currents is thus a direct strategy to enhance magnetization switching efficiency. However, traditional strong SOC materials exhibit a positive correlation between the magnitude of the spin‐polarization current generated by the spin Hall effect and spin Hall conductivity. Therefore, a large spin Hall effect inevitably leads to high resistivity, which is undesirable for the low‐power consumption requirements of practical applications.

**FIGURE 1:**
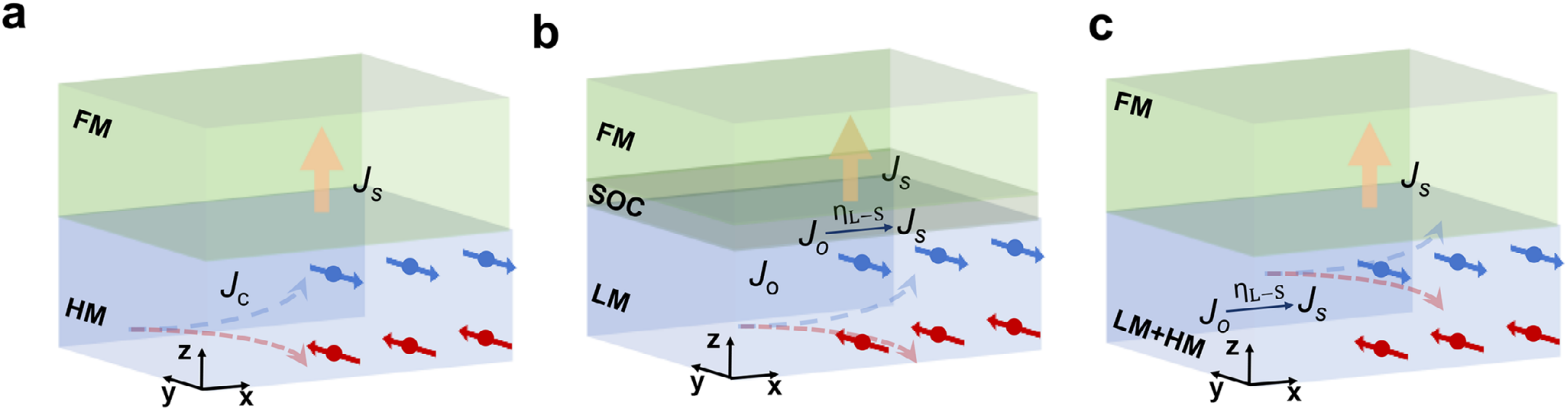
Charge current to Spin current conversion. (a) Schematic of the ST system. Spin current generated from the heavy metals (HMs) due to the spin Hall effect (SHE) is injected into the adjacent ferromagnetic (FM) layer, exerting the SOT. (b) Schematic of the OT system. Orbital current originated from the light materials (LMs) due to the orbital Hall effect (OHE) is injected into the adjacent FM layer, then the orbital current is converted to spin current in the FM layer due to the orbital‐to‐spin conversion. (c) Hybrid of ST and OT system. Alloys of light metals with OHE and heavy metals with SHE as spin source layers.

Recently, orbital torque (OT) has emerged as a promising way toward highly efficient electrically‐driven magnetization switching. According to theoretical studies, the orbital Hall conductivity is typically several times, or even an order of magnitude, larger than the spin Hall conductivity in many materials [15, 16, 17]. As shown in Figure 1b, the orbital current can be generated in light materials (LMs) via the orbital Hall effect (OHE) or orbital Rashba‐Edelstein effect (OREE), and subsequently converted into a spin current either within the FM layer with SOC or within an inserted SOC layer between the LM layer and FM layer. The strength of the resulting orbital torque can be scaled with the product of orbital‐to‐spin conversion efficiency and orbital Hall angle (***η***_L‐S_· ***θ***_OHE_) [18]. In such OT systems, the properties of both the orbital Hall materials and the SOC materials play indispensable roles. Previous works have used magnetic materials with strong SOC [18, 19, 20, 21, 22, 23, 24, 25] (e.g., Ti/Ni, Zr/CoPt) or inserted an ultrathin SOC layer [26, 27, 28, 29, 30] (e.g., CoO_x_/Pt/Co, Ta/Pt/TbIG) to convert the orbital currents into spin currents and achieve electrically‐driven perpendicular magnetization switching. However, in these schemes, once the OHE and SOC materials are determined, it is difficult to modulate the magnitudes of ***η***_L‐S_ and ***θ***_OHE_, which hinders the achievement of efficient and tunable charge‐to‐spin conversion.

In this work, we design NiNb alloys by integrating niobium (Nb), which exhibits strong OHE, with nickel (Ni), which possesses strong SOC [31]. By continuously adjusting the Ni/Nb ratio, we can control the relative magnitudes of the OHE (***θ***_OHE_) and SOC (***η***_L‐S_) and generate large spin currents through the synergistic effect of OHE and SOC. The generated torque allows the current density required for switching perpendicular magnetization to be reduced by an order of magnitude compared with conventional heavy metal (platinum or tantalum) materials. Our work highlights the dominant role of extrinsic disorder scattering in unconventional OHE systems. The ab‐initio calculation results indicate that increasing the Ni content in NiNb alloys enhances the SHE strength while suppresses the OHE strength. This opposing trend collectively accounts for the peak in the charge‐spin conversion efficiency observed at the ratio of Ni_50_Nb_50_.

## 2 Experimental Section

The NiNb/Ni_81_Fe_19_(Py) and NiNb/CoTb thin films were deposited on Si/SiO_2_ substrates by magnetron sputtering at room temperature. The magnetic anisotropy of the heterostructure was confirmed by magneto‐optic Kerr effect (MOKE). As presented in Figure S1, the typical NiNb/Py sample exhibits in‐plane magnetic anisotropy, whereas the typical NiNb/CoTb sample exhibits perpendicular magnetic anisotropy. To quantify the current‐induced spin torque, we employed spin torque‐induced ferromagnetic resonance (ST‐FMR) measurement in NiNb/Py devices (Figure 2a). This technique detects a DC mixing voltage (*V*_mix_) generated by the interaction of the microwave current (*I*_rf_) and the oscillating magnetization dynamics of the Py layer via magnetoresistance. The ST‐FMR signal comprises two components: a symmetric Lorentzian (*V*_s_) and an antisymmetric Lorentzian (*V*_a_) (Figure 2b) [9]:

(1) $$V_{mix\;}=V_{offset}+V_{s}\frac{\Delta H^{2}}{{\left(H-H_{r}\right)}^{2}+\Delta H^{2}}+V_{a}\frac{\Delta H(H-H_{r})}{{\left(H-H_{r}\right)}^{2}+\Delta H^{2}}$$

where *V*_offset_ is the offset voltage, *H*_r_ is the resonant field, and Δ*H* is the line width. As shown in the inset of Figure 2c, the *I*_rf_ was applied to the device with a frequency of 8 GHz and power amplitudes from 15 to 35 dBm (1 mW = 10^0.1*1 dBm^). When the microwave power is below 30 dBm (1000 mW), the signals of both *V*_s_ and *V*_a_ are in the linear region with respect to the power, so subsequent tests were carried out with the power kept below 30 dBm. The resonance field (*H*_r_) dependence of the microwave frequency (*f*) is shown in Figure 2c. The effective demagnetization field is calculated according to the Kittel equation for in‐plane ferromagnetic films [9, 32]:

(2) $$f=\frac{\gamma }{2\pi }\;\sqrt{\left(4\pi M_{eff}+H\right)H}$$

where 4π*M*_eff_ is effective demagnetization field, *H* is external magnetic field. The effective demagnetization field (4𝜋*M*_eff_), 7924 Oe, is consistent with the saturation magnetization (4𝜋*M_s_*), 9625 Oe, measured by vibrating sample magnetometry (VSM); see the Supporting Information.

**FIGURE 2:**
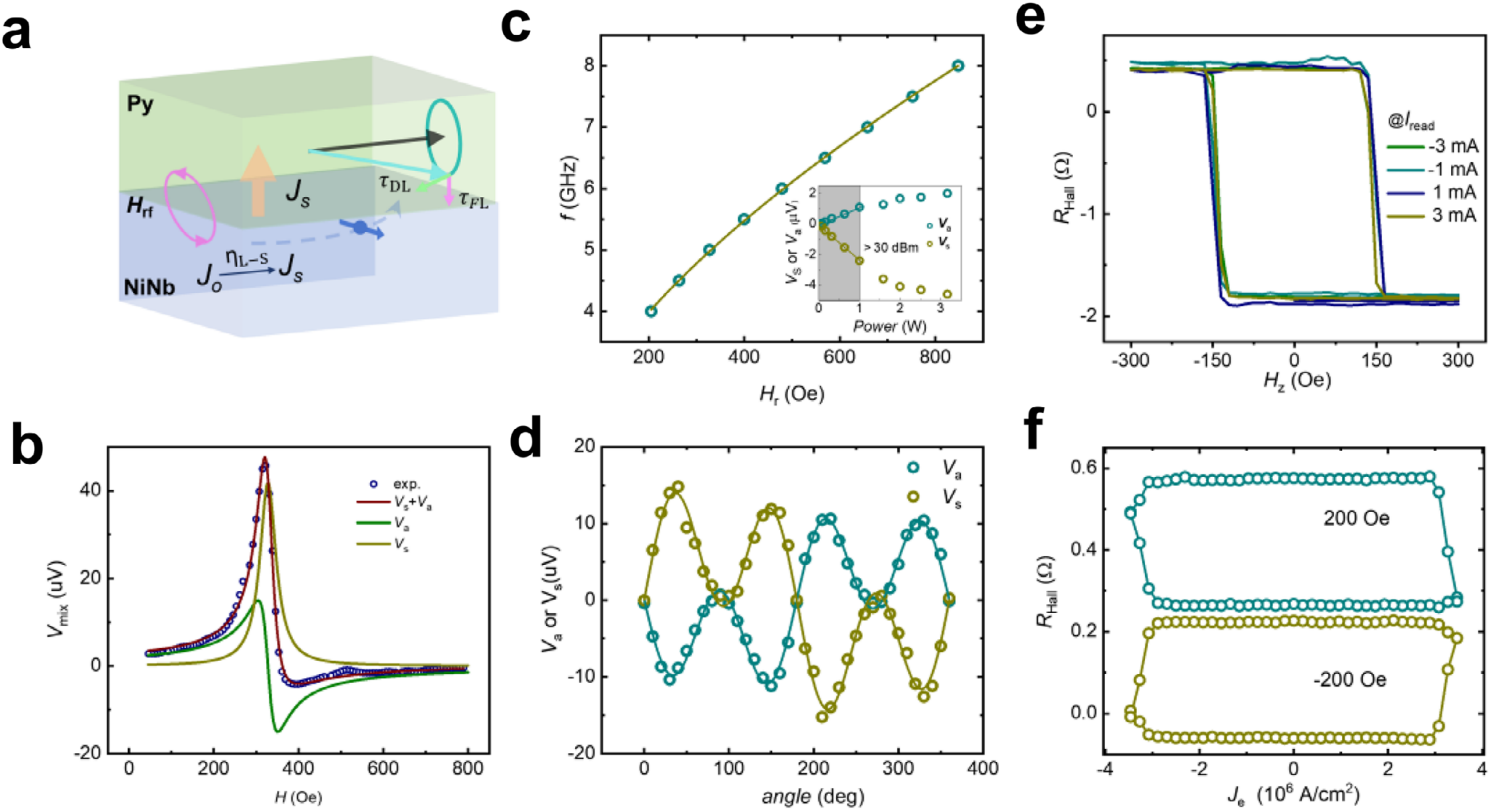
(a–d) ST‐FMR measurement of Ni_50_Nb_50_(10)/Py(8)/capping (nm). (a) Schematic of the ST‐FMR configuration and torques (τ_DL_ andτ_FL_) induced by the injected microwave current (*I*_rf_). (b) The mixing voltage (*V*_mix_) of the NiNb/Py device at 6 GHz and *φ*_H_ = 45°. Dots represent experimental data and solid lines represent the fitted curves, where the *V*_mix_ can be decomposed into symmetric Lorentzian (*V*_s_) and antisymmetric Lorentzian (*V*_a_) components. (c) *H*_r_ dependence of the microwave frequency. Inset shows *V*_s_ and *V*_a_ signals at different microwave power. The grey region indicates that the *V*_s_ and *V*_a_ signals are in the linear region of microwave power. (d) *V*_a_ and *V*_s_ voltage signals as a function of *φ*_H_. (e,f) Electrical transport measurements of Ni_50_Nb_50_(10)/CoTb(8)/capping device. (e) The AHE loop at different dc readings current. (f) Current‐induced magnetization switching with the fixed in‐plane magnetic field *H*_ext_ = +200 Oe or −200 Oe.

The angle (*φ*_H_) dependence of the *V*_mix_ is obtained by rotating the sample plane relative to the magnetic field direction. The amplitude of the damping‐like (DL) and field‐like (FL) torques of spin current can be separated by fitting the *φ*_H_ dependence with Equations (3) and (4) (Figure 2d) [13, 33]:

(3) $$V_{s}=S_{y}^{\mathrm{DL}}\;\cos\varphi_{H}\sin2\varphi_{H}$$

(4) $$V_{a}=A_{y}^{\mathrm{FL}}\;\cos\varphi_{H}\sin2\varphi_{H}$$

where$\;A_{y}^{\mathrm{DL}}$ and $S_{y}^{\mathrm{DL}}$ represent the amplitude of the DL (FL) torque generated by the *y*‐directional spin polarization components. Furthermore, the corresponding ST efficiency [34],

(5) $$\varsigma_{y}^{\mathrm{ST}}=\frac{S_{y}^{\mathrm{DL}}}{\;A_{y}^{\mathrm{DL}}}\;\frac{e\mu_{0}M_{s}t_{Py}t_{NiNb}}{\hbar }\sqrt{1+\frac{M_{eff}}{H_{r}}}$$

Here, e, *t_Py_*, *t_NiNb_*, ℏ, and *H_r_* are the elementary charge, thickness of Py, thickness of NiNb, the reduced Planck constant, and the resonance field of Py at 8 GHz, respectively. Significantly, in the Ni_50_Nb_50_/Py sample, the ST efficiency reaches a value of $\varsigma_{y}^{\mathrm{ST}}=-1.7$ (even $|\varsigma_{y}^{\mathrm{ST}}|$> 1), which is significantly larger than that of the conventional heavy metals (Pt and Ta), as shown in the Note S9.

To further investigate the efficiency of the magnetization switching through the Nb (orbital Hall materials, OHM) and Ni (spin Hall materials, SHM), we prepared the film stack of NiNb(10)/CoTb(8)/SiN(3) (thickness in nanometers). The square anomalous Hall effect (AHE) loops indicate good PMA property of the NiNb/CoTb sample, as shown in Figure 2e. The current‐induced magnetization switching was performed under an in‐plane *H*_ext_ along the current direction, as shown in Figure 2f. Although the resistivity of the NiNb alloy varies monotonically with composition, it remains much lower than that of the CoTb layer (Table S1). Notably, the critical switching current density is 3.24 × 10^6^ A/cm^2^, which is an order of magnitude lower than conventional Pt/CoTb (∼4 × 10^7^ A/cm^2^) and Ta/CoTb (∼1 × 10^7^ A/cm^2^) systems, and close to that of the topological insulator Bi_2_Se_3_/CoTb (∼3 × 10^6^ A/cm^2^) [35]. The reduced switching ratio in the CoTb‐based devices is likely associated with the ferrimagnetic nature and multidomain switching behavior of the CoTb layer, as also reported in other ferrimagnetic systems [36, 37]. Such multidomain switching behavior is further supported by our MOKE measurements. To verify the switching capability of NiNb‐based devices, we also measured current‐induced magnetization switching in NiNb/Pt/Co/Pt control samples. As shown in Figure S20, nearly full magnetization switching was achieved, its critical switching current is slightly higher than that of NiNb/CoTb devices when the density is higher. In the NiNb/Pt/Co/Pt sample, a torque efficiency comparable to that in the NiNb/CoTb sample was observed, while the slightly lower critical switching current density in the CoTb‐based sample can be attributed to the negative exchange interaction in the ferrimagnetic layer [38, 39] (Figure S19). Furthermore, based on a comparison of the SOT‐driven perpendicular magnetization reversal characterization of the samples, the role played by the SOC of the rare earth element Tb in CoTb can be considered negligible in this system. These results suggest that the synergistic effect of the orbital Hall effect of Nb and the strong SOC of Ni in NiNb alloys can efficiently convert charge currents into spin currents for the perpendicular magnetization switching.

Subsequently, in order to better understand the process of charge current through the synergistic action to generate spin current in NiNb alloys, we prepared NiNb alloys with different compositions (Ni_40_Nb_60_、Ni_45_Nb_55_、Ni_55_Nb_45_, and Ni_60_Nb_40_) to modulate the strength of the orbital Hall (***θ***_OHE_) and spin‐orbit coupling (***η***_L‐S_) in the system by the stoichiometric ratio modulation. First, we characterized the resistivity vs. temperature in NiNb alloys with different stoichiometric ratios using the van der Pauw method, as shown in Figure 3a. The resistivity of NiNb alloys decreases with increasing temperature, consistent with previous studies [40]. At room temperature, the resistivity exhibits a monotonic increase with increasing Nb content, as shown in Figure 3b. The charge‐to‐spin conversion efficiency of these NiNb alloys were also measured by ST‐FMR (see Figure S2). Although the resistivity of NiNb varies with composition, the spin‐torque efficiency was determined by ST‐FMR using the conventional V_S_/V_A_ method, and the current‐shunting factor between the NiNb and NiFe layers cancels out in V_S_/V_A_ [9]. Therefore, the current‐shunting effect between the NiNb and NiFe layers is not expected to significantly affect the extracted spin‐torque efficiency. In a classical SOC system, the conventional trend is satisfied: as resistivity increases, the SOT efficiency also increases, which consistent with the scatting nature of the spin current generation. However, in NiNb alloys, the charge‐to‐spin conversion efficiency ($\varsigma_{y}^{\mathrm{ST}}$) shows a non‐monotonic variation with resistivity, as summarized in Figure 3c. In Ni‐rich region (Ni:Nb > 50:50), the $\varsigma_{y}^{\mathrm{ST}}$ increase with the increase of resistivity, whereas in Nb‐rich region (Ni:Nb < 50:50), the $\varsigma_{y}^{\mathrm{ST}}$ decrease with the increase of resistivity. The maximum of $\;\varsigma_{y}^{\mathrm{ST}}$= −1.7 is obtained at the stoichiometric ratio of Ni_50_Nb_50_. This “non‐monotonic” behavior between $\varsigma_{y}^{\mathrm{ST}}$ and resistivity indicates that neither the SHE nor the OHE alone can account for this phenomenon [22]. Instead, it originates from their synergistic interplay in NiNb alloys. This synergistic effect of spin–orbit coupling and the OHE in NiNb alloys not only yields a very high charge‐to‐spin conversion efficiency, but also provides a new mechanism for enhancing spin generation efficiency.

**FIGURE 3:**
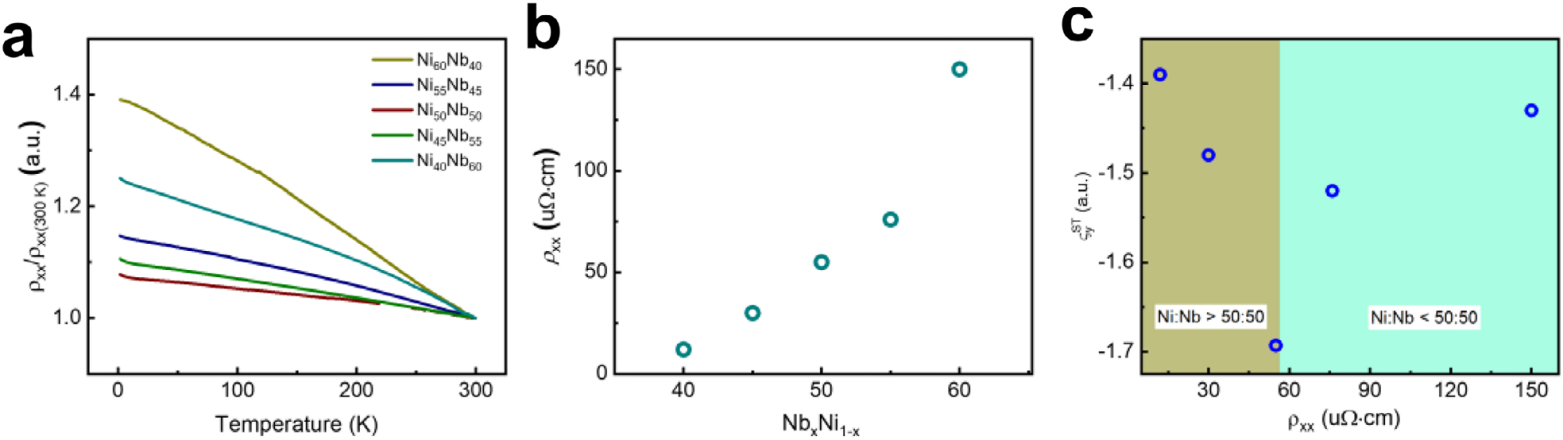
(a) R‐T curves of NiNb alloys with different compositions. (b) The room temperature resistances of NiNb alloys with different compositions. (d) Dependence of charge‐to‐spin efficiency on resistivity. Dark yellow region corresponding to Ni‐rich alloys where the spin Hall effect dominates, and dark green region corresponding to Nb‐rich alloys where the orbital Hall effect dominates.

In order to better study the synergy between orbital and spin in NiNb alloys, We prepared samples of Ni_50_Nb_50_(*t*)/Pt(1.5)/Co(0.7)/Pt(1.5) and Ni_50_Nb_50_(*t*)/Ta(1) /Pt(1.5) /Co(0.7)/Pt(1.5) with different Ni_50_Nb_50_ layer thicknesses, where t = 4, 6, 8, 10, 15, and 20 nm. We characterized the SOT efficiency of this system using the second‐harmonic technique. In both systems, the charge‐to‐spin conversion efficiency increased with increasing Ni_50_Nb_50_ thickness, and as the charge‐to‐spin conversion efficiency increased, the critical switching current density gradually decreased (as shown in Figure S13), further demonstrating that the large spin current generated through efficient orbital‐spin synergy can effectively control perpendicular magnetization switching. By fitting the thickness‐dependent SOT efficiency (***𝜎***_eff_) using the equation: $\sigma_{eff}=\theta_{SH}^{eff}\;\left[1-sech(\frac{t}{\lambda_{L(S)}})\right]$[41, 42], we extracted $\theta_{SH}^{eff}$ and the orbital (spin) diffusion length (λ_*L*(*S*)_). The extracted *𝜆*_L_ is ∼6.7 ± 1.2 nm for the Ni_50_Nb_50_(t)/ Pt(1.5)/Co(0.7)/Pt(1.5 nm) and 5.7 ± 0.4 nm for the Ni_50_Nb_50_(t)/Ta(1)/Pt(1.5) /Co(0.7)/Pt(1.5 nm), longer than the *𝜆*_S_ the Ta. This is consistent with previous reports, where the orbital current penetrates a longer distance in solids compared to spin current [43, 44, 45].

Ultimately, we found that NiNb alloys and NiNb/NiFe heterojunctions exhibited small changes in resistivity and remained in a high‐resistivity metallic state when the temperature was lowered. However, their charge‐to‐spin efficiency increased by up to a factor of 2.37 at room temperature, which directly indicates the enhanced role of the orbital Hall effect at low temperatures. This leads to a significant improvement in their efficiency.

### 2.1 Model and Simulations

To elucidate the synergistic interaction between orbital and spin materials in spin–orbit hybrid systems that enables highly efficient charge‐spin conversion, the DFT calculations are conducted. We modelled the NiNb alloys with unit cells comprising a fixed Ni component and various Nb components (Ni_4_Nb_8_, Ni_4_Nb_6_, Ni_4_Nb_4_, Ni_4_Nb_3,_ and Ni_4_Nb_3_), as illustrated in Figure 4a. The corresponding DFT energy band diagrams are shown in Figure 4b. In such a hybrid system, where the OHE and SHE act synergistically, the efficiency of converting a charge current into a spin current is governed by two main mechanisms: (1) The spin current generated by the SHE directly; (2) The orbital current generated by the OHE is first converted into a spin current by the SOC. Therefore, the strengths of spin Hall conductivity (SHC), orbital Hall conductivity (OHC), and orbital hybridization (which contributes to the proposed ***η***_L‐S_) are all crucial to produce the final charge‐to‐spin conversion.

**FIGURE 4:**
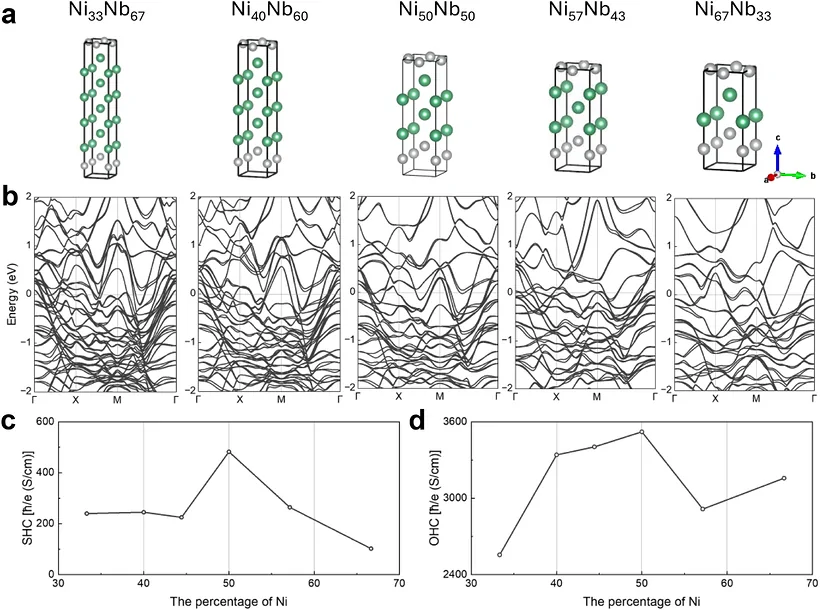
(a) The schematic structure of NiNb designed with different composition ratios. (b) Band structure of NiNb structure with different composition ratios obtained from ab‐initio calculation. (c,d) Spin Hall conductance (c) and orbital Hall conductivity (d) coefficients for NiNb alloys with different Ni contents.

Given this, it is found that as the Ni content changed systematically, the strengths of both the spin Hall effect and the orbital hybridization between Ni and Nb can be enhanced. As a result, the spin and orbital Hall conductivities for the various Ni compositions are summarized in Figure 4c and Figure 4d. Both the SHC and OHC exhibit their maximum values at the 1:1 stoichiometric ratio of Ni to Nb. The experimentally extracted effective spin/orbital Hall conductivity exhibits a composition‐dependent trend consistent with the theoretical calculations, increasing initially and then decreasing with increasing Nb content (Figure S3). Moreover, the electronic density of state (DOS) of Ni and Nb differs across the energy bands (details see Figure S10), and the strongest orbital hybridization is observed in the Ni_4_Nb_4_ structure when the DOS of Ni and Nb are closest at the Fermi surface. These results indicate that the SHC, OHC, and ***η***_L‐S_ are all enhanced by composition mixing and the maximum values at the 1:1 stoichiometric ratio, which agree well with the experimental trend shown in Figure 3c. The calculated results emphasize the synergistic effect of the orbital Hall effect and SOC, which can produce a very high charge‐spin‐conversion efficiency. Furthermore, the calculation provides an efficient pathway for optimizing spin conversion efficiency in material systems where the orbital Hall and spin Hall effects operate synergistically.

### 2.2 Methods

The studied NiNb/Py and NiNb/CoTb samples were deposited on a thermally oxidized Si/SiO_2_ substrate using magnetron sputtering at room temperature. The base pressure of the vacuum chamber is better than 1 × 10^−8^ Torr. The multilayers were deposited at an Ar pressure of 3 × 10^−3^ Torr by DC magnetron sputtering, and NiNb alloys were prepared by co‐sputtering Ni and Nb targets. A capping layer (3 nm Al_2_O_3_) was used to protect the samples from oxidation. A combination of photolithography and ion‐beam etching was used to pattern the NiNb(10)/CoTb(8)/capping samples to the Hall bar devices (channel size: 20 µm × 100 µm) and the NiNb(10)/Ni_81_Fe_19_(8)/capping samples to ST‐FMR devices (strip size: 20 µm × 100 µm), respectively. Then the Ti(5)/Cu(100) (thickness in nanometers) top electrodes were deposited by DC magnetron sputtering. Current‐induced magnetization switching measurements were conducted by utilizing a dual‐channel Keithley 2636B source meter. A pulse current *I*_dc_ with a width of 3 ms and varying amplitude was applied to the Hall bar device as the writing current, and the Hall resistance was measured after each writing pulse current with a low reading current of 1 mA. The amplitude of the write pulse current was varied to obtain a complete *R*‐*I* loop. ST‐FMR measurements were performed at room temperature to estimate the spin–orbit torque (SOT) efficiency. The radio‐frequency (rf) microwave current modulated at 777 Hz was applied to the device at a fixed frequency ranging from 6 to 18 GHz with a power P = 25 dBm, and a mixing dc voltage was measured by a lock‐in amplifier. A bias tee was used to divide RF and dc signals.

## 3 Conclusion

In this work, we achieved an efficient charge‐to‐spin conversion ratio of −1.4–−1.7 through the combined action of the orbital and spin Hall effects in NiNb alloys. The generated spin currents can efficiently switch the perpendicular magnetization, with a critical switching current density an order of magnitude lower than that of the conventional heavy metals such as Pt and Ta, and its orbital diffusion length reaches 5.7–6.7 nm, which is far greater than the spin diffusion length of traditional heavy‐metal systems. Furthermore, the non‐monotonic dependence of the charge‐to‐spin conversion efficiency on resistance offers a new design pathway to improve spin generation that goes beyond the conventional spin Hall effect. Our findings indicate that alloys combining spin Hall and orbital Hall materials hold great potential for the development of low‐power, high‐density spintronic devices.

## Author Contributions

**Jing Zhou**: methodology, investigation, formal analysis, Writing – review and editing. **Zhaowei Zhang**: investigation, data curation, formal analysis. **Jiali Chen**: validation, writing – review and editing, data curation. **Jinkui Zhao**: investigation, formal analysis, data curation. **Yibo Fan**: writing – original draft, methodology. **Wei Jiang**: methodology, investigation, validation, writing – review and editing, formal analysis, funding acquisition. **Chuangwen Wu**: methodology, software, investigation, validation, formal analysis, writing – original draft, writing – review and editing, conceptualization, data curation. **Chuantong Ren**: investigation, validation, data curation. **Zonghao Deng**: investigation, data curation. **Rongce Sun**: investigation, data curation. **Zengxin Wei**: investigation, data curation. **Jing Zhang**: investigation, formal analysis, data curation. **Xingen Zheng**: investigation, data curation. **Jingfeng Li**: investigation, formal analysis, data curation. **Hao Wu**: conceptualization, methodology, software, data curation, investigation, validation, formal analysis, supervision, funding acquisition, visualization, writing – review and editing, writing – original draft, project administration, resources. **Haibo Ke**: investigation, funding acquisition, methodology, writing – review and editing, formal analysis, validation. **Weihua Wang**: funding acquisition, writing – review and editing, visualization, resources.

## Conflicts of Interest

The authors declare no conflicts of interest.

## Supporting information




**Supporting File**: advs76805‐sup‐0001‐SuppMat.docx.

## Data Availability

The data that support the findings of this study are available from the corresponding author upon reasonable request.
