# Supplementary material for: Efficient Current‐Driven Perpendicular Magnetization Switching Through the Synergy of the Orbital and Spin Hall Effects
Source: Adv Sci (Weinh). 2026 Jul 28:e76805. Online ahead of print. doi: 10.1002/advs.76805 (PMC13410799; doi:10.1002/advs.76805)
Supplement: Supplementary file 1 — Supporting File: advs76805‐sup‐0001‐SuppMat.docx. [file ADVS-9999-e76805-s001.docx]

**Efficient current-driven perpendicular magnetization switching through thesynergy of the orbital and spin Hall effects**

Chuangwen Wu^#^, Jing Zhou^#^, Yibo Fan^#^, Jiali Chen^#^, Rongce Sun, Zonghao Deng, Zengxin Wei, Chuantong Ren, Xingen Zheng, Zhaowei Zhang, Jing Zhang, Jingfeng Li, Jinkui Zhao, Wei Jiang^*^, Haibo Ke^*^, Hao Wu^*^, Weihua Wang

Dr. C. W. Wu, Dr. J. Zhou, Dr. Y. B. Fan, R. C. Sun, Z. H. Deng, Dr. Z. X. Wei, C. T. Ren, Dr. X. G. Zheng, Dr. J. Zhang, Dr. J. F. Li, Prof. H. B. Ke, Prof. H. Wu, Prof. W. H. Wang

*Dongguan Institute of Materials Science and Technology, Chinese Academy of Sciences, Dongguan, Guangdong 523808, China*

*Songshan Lake Materials Laboratory, Dongguan, Guangdong 523808, China*

J. Chen, Prof. W. Jiang

*Key Laboratory of Advanced Optoelectronic Quantum Architecture and Measurement (MOE), School of Physics, Beijing Institute of Technology, China*

Dr. C. W. Wu, Dr. Z. W. Zhang, Prof. J. K. Zhao

*School of Physical Sciences, and Department of Physics, School of Sciences, Great Bay University, Dongguan 523000, China*

**contents**

[Note 1. In-plane magnetic measurement for the NiNb/Py sample 3](#_Toc233050874)

[Note 2. ST-FMR of NiNb/Py/SiN with different compositions 3](#_Toc233050875)

[Note 3. PMA of NiNb/CoTb/SiN with different compositions 5](#_Toc233050876)

[Note 4. Loop-shift measurement 6](#_Toc233050877)

[Note 5. Second harmonic Hall measurement of torque efficiency. 7](#_Toc233050878)

[Note 6. SOT Efficiency for Different interlayer Thicknesses 10](#_Toc233050879)

[Note 7. SOT Efficiency for Different Ni_50_Nb_50_ Layer Thicknesses 12](#_Toc233050880)

[Note 8. Current-driven magnetization switching 13](#_Toc233050881)

[Note 9. SOT-Driven perpendicular Magnetization Switching Ratio 18](#_Toc233050882)

[Note 10. Comparison of Samples from Traditional Heavy Metal Systems 19](#_Toc233050883)

[Note 11. Temperature-dependent charge-spin conversion efficiency 21](#_Toc233050884)

[Note12. Theoretically calculated orbital hybridization of Ni and Nb 23](#_Toc233050885)

# Note 1. In-plane magnetic measurement for the NiNb/Py sample

The in-plane magnetic hysteresis loop for the Ni_50_Nb_50_/Py(8)/SiN(5) sample was measured using the vibrating sample magnetometer (VSM) module of a superconducting quantum interface device (SQUID). The loop exhibits a saturation magnetization value of 766 emu/cc for the Py layer as shown in Fig.S1(a). The hysteresis lines of the grown Py layer on the NiNb alloy of different compositions, measured using in-plane magneto-optical Kerr microscopy, are shown in Fig. S1b.


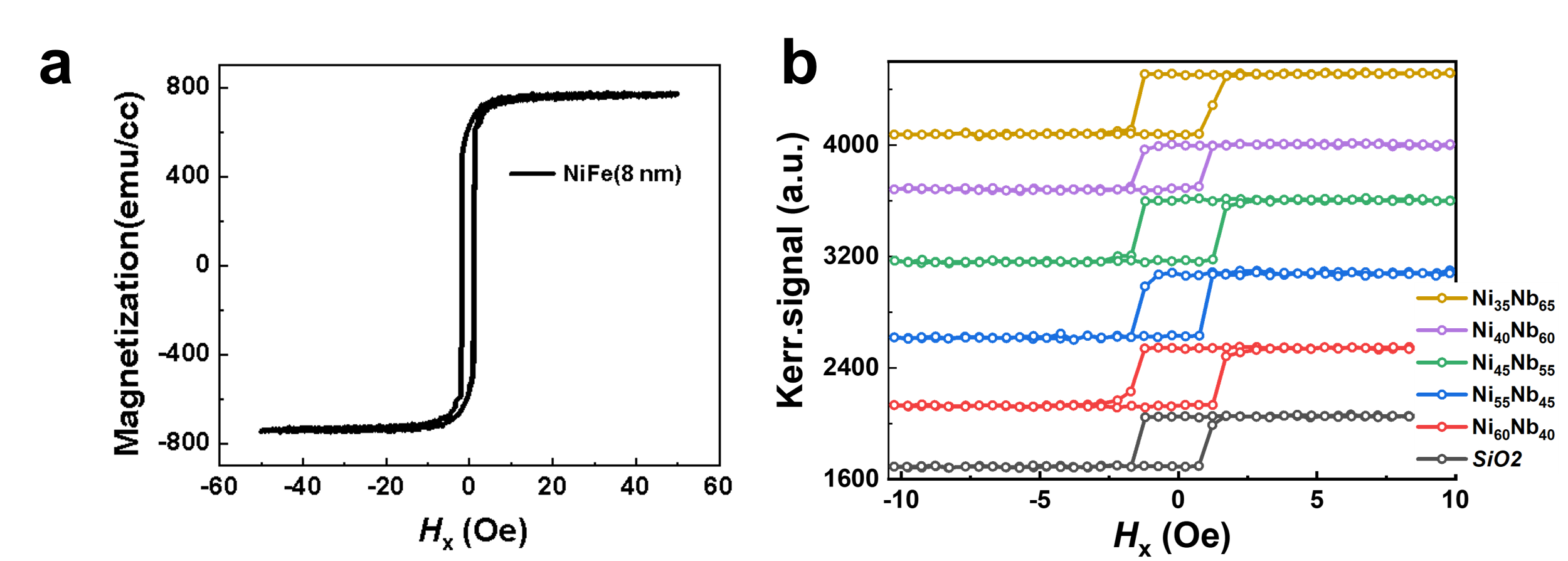


Figure S1 (a) M-H loops of the NiNb/Py(8)/SiN(5) sample along the in-plane magnetic. (b) MOKE loop of the Ni_x_Nb_100-x_/Py(8)/SiN(5) alloy of different compositions.

# Note 2. ST-FMR of NiNb/Py/SiN with different compositions

The in-plane Py layer (Ni_81_Fe_19_) was grown on these amorphous NiNb alloy samples (Ni_35_Nb_65_、Ni_40_Nb_60_、Ni_45_Nb_55_、Ni_55_Nb_45_、Ni_60_Nb_40_ and Ni_65_Nb_35_), and then the samples were prepared into devices by micro-nanofabrication, and then the spin Hall efficiency was investigated by using the spin-torque ferromagnetic resonance technique using the turning angle. We defined the microwave current direction parallel to the magnetic field direction as 0°, and then measured its ferromagnetic resonance spectra at every 10° turn angle, and its symmetric and antisymmetric signals under each angle (*φ*) were extracted. In NiNb alloys, only the conventional spin-polarised currents in the y-direction were present. The magnitude of the spin Hall angle at each of its compositions was first investigated using angle-dependent spin moment ferromagnetic resonance at its room temperature, as shown in Fig. S2.


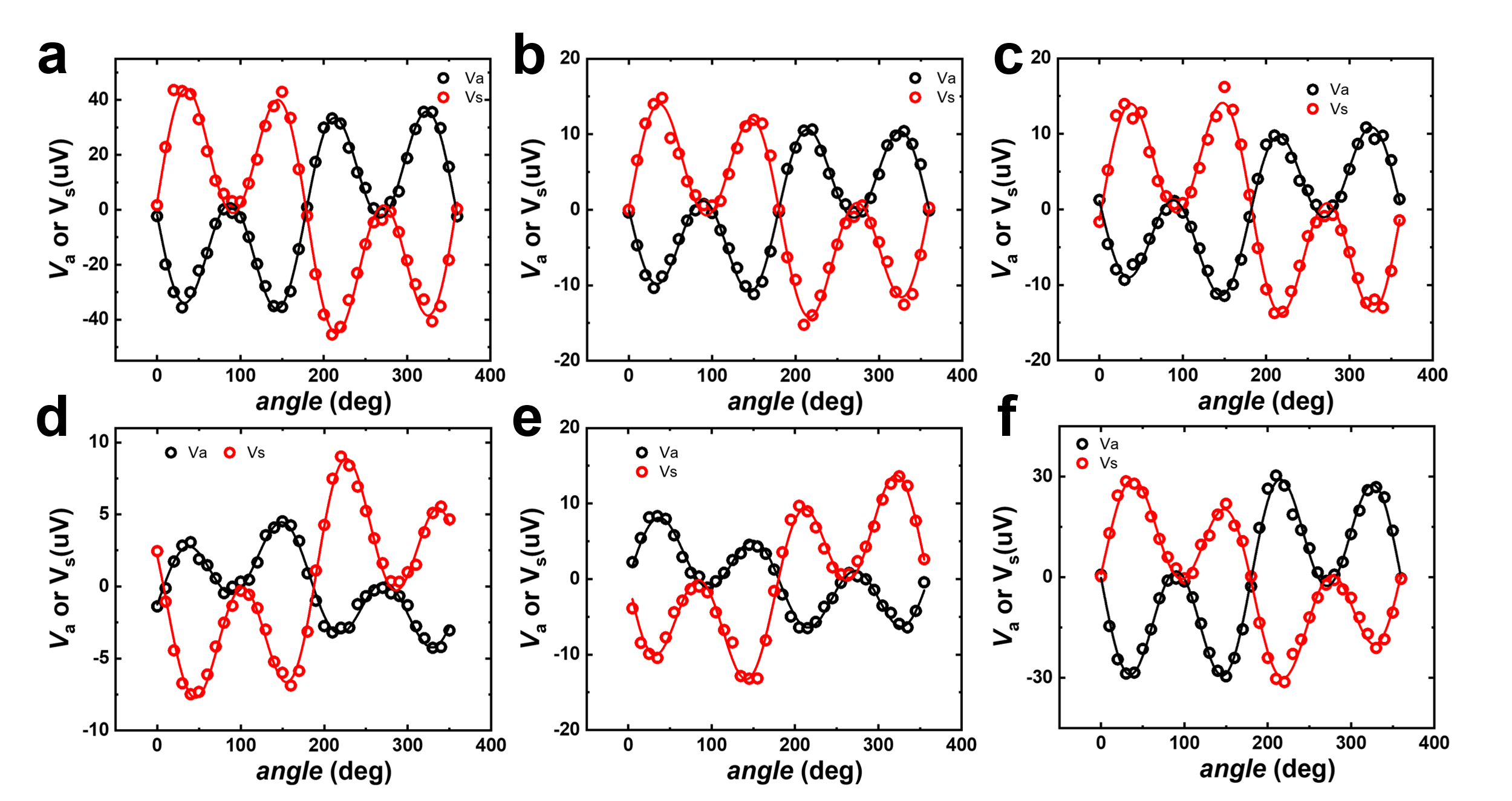
 Figure S2. Spin torque ferromagnetic resonance measurements. Va and Vs voltage signals as a function of *φ* for NiNb alloys with different compositions (a) Ni_35_Nb_65_/Py/SiN, (b) Ni_40_Nb_60_/Py/SiN, (c) Ni_45_Nb_55_/Py/SiN, (d) Ni_55_Nb_45_/Py/SiN, (e) Ni_60_Nb_40_/Py/SiN, and (f) Ni_65_Nb_35_/Py/SiN.

The experimentally extracted effective spin/orbital Hall conductivity ($\sigma_{\mathrm{eff}}=\theta_{\mathrm{SH}}\times\sigma_{c}\times\frac{\hbar}{2e}$) exhibits a composition-dependent trend consistent with the theoretical calculations, increasing initially and then decreasing with increasing Nb contents (Supporting Figure S3).


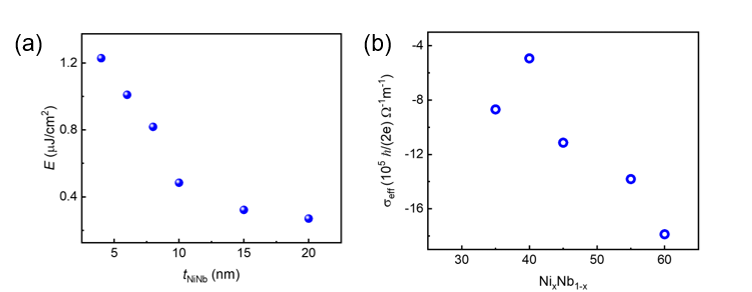


Figure S3 Spin/orbital Hall conductivities of NiNb/CoTb devices with different compositions.

# Note 3. PMA of NiNb/CoTb/SiN with different compositions

MOKE-loop of Ni_50_Nb_50_(10)/CoTb(10)/SiN(5 nm) heterojunctions and the dynamics of the magnetization switching of the CoTb layer under the magnetic field, as shown in Fig. S4. And Fig. S5 illustrates the anomalous Hall loops and MOKE loops of NiNb(10)/CoTb(10)/SiN(5 nm) for NiNb alloys of different compositions.


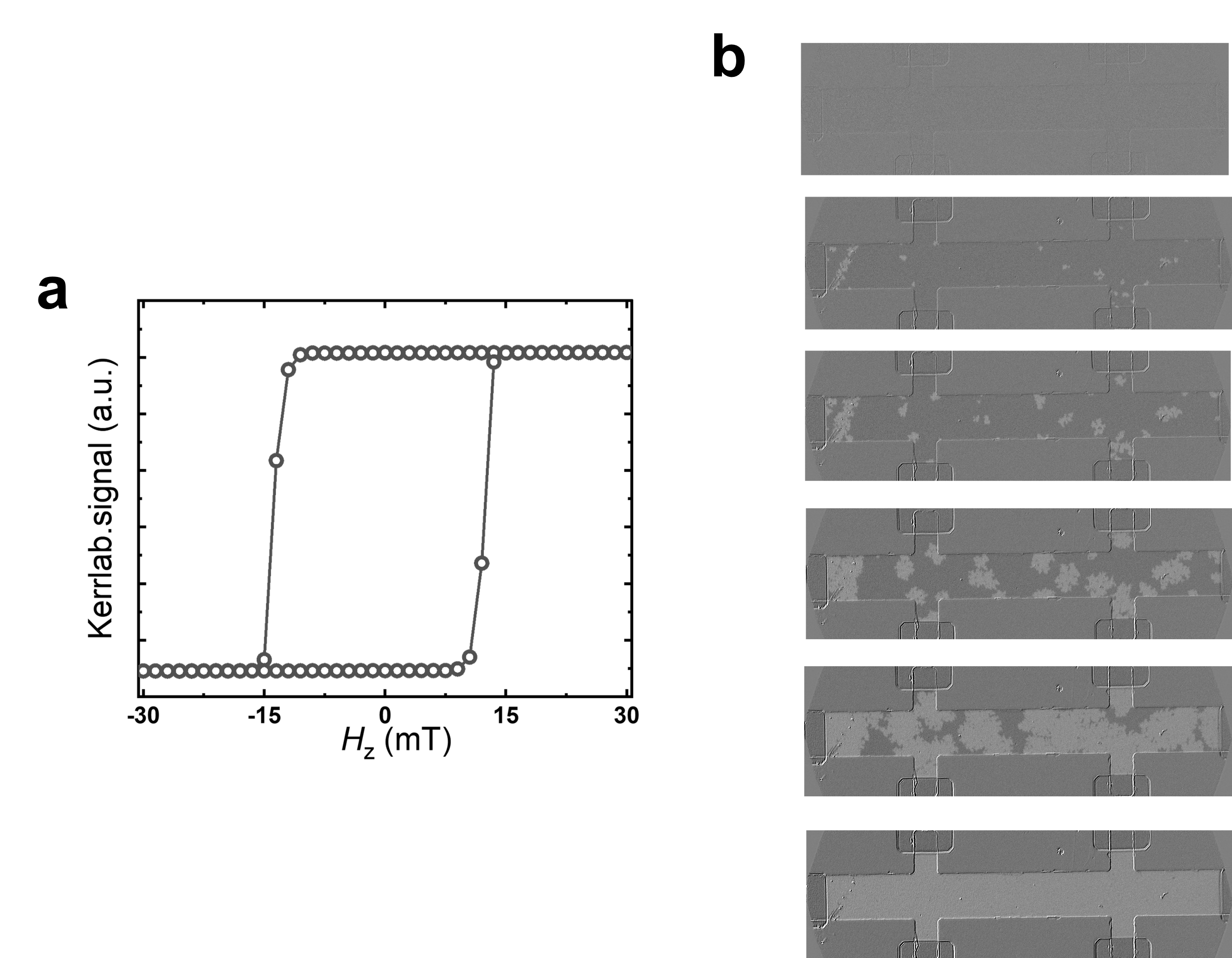


Figure S4. (a) MOKE-loop of Ni_50_Nb_50_(10)/CoTb(10)/SiN(5 nm). (b) Dynamics of the magnetization switching of the CoTb layer under the magnetic field.


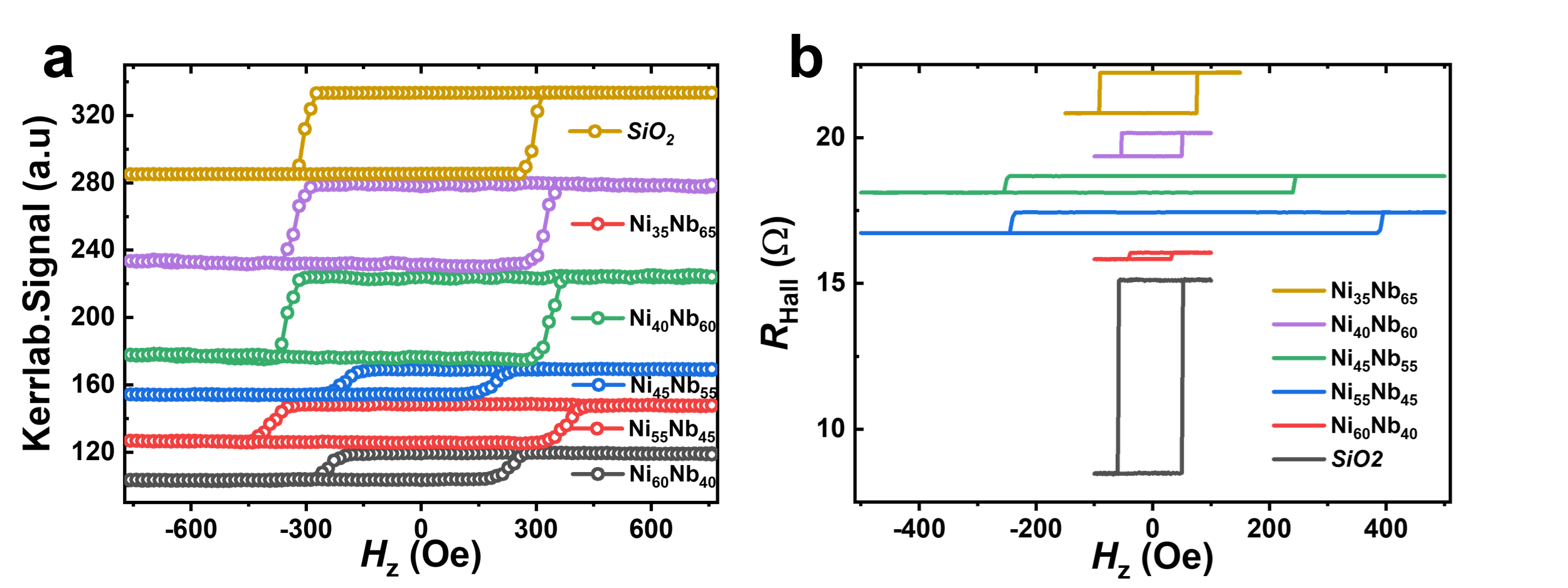


Figure S5. The MOKE loops (a) and anomalous Hall loops (b) of NiNb(10)/CoTb(10)/SiN(5 nm) for NiNb alloys of different compositions.

# **Note 4. Loop-shift measurement**

Next, we use the hysteresis loop shift method to characterize the DMI along current directions. We measure the hysteresis loop of the anomalous Hall signal *R*_xy_ (this is, the *M*_z_ component of the top perpendicular CoTb layer) versus the out-of-plane field *H*_z_ in the presence of d.c. current *I*_dc_ (along x) and in plane field (along x), as shown in Fig. S6.


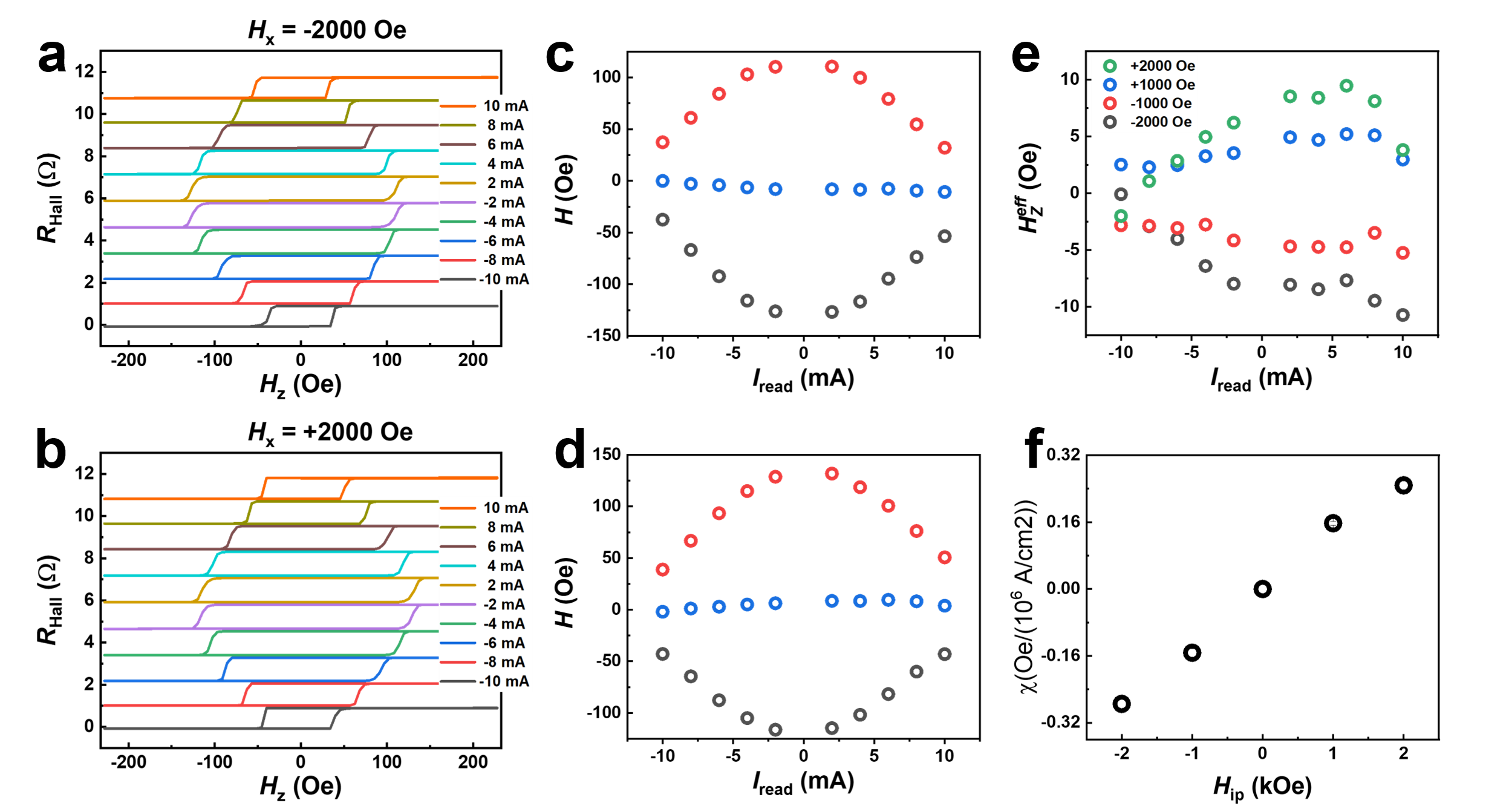


Figure S6. (a-b) Anomalous Hall loop shift for different DC reading current. (a) *H*_x_ = -2000 Oe. (b) *H*_x_ = +2000 Oe. (c-d) The out-of-plane switching fields *H*_SW_ of a Ni_50_Nb_50_(10)/CoTb(10)/SiN(5 nm) Hall-bar sample as functions of *I*_dc_. (c) *H*_x_ = -2000 Oe. (d) *H*_x_ = +2000 Oe. (e) The effective field $H_{z}^{eff}$ as a function of *I*_dc_ for different in-plane magnetic fields. (f) Current-induced effective field per current density χ of Ni_50_Nb_50_(10)/CoTb(10)/SiN(5 nm) as a function of in-plane bias field *H*_x_.

# Note 5. Second harmonic Hall measurement of torque efficiency.

Second harmonic Hall measurement is widely used to evaluate the SOT effective field. By applying ac. electric field, alternating torque is exerted on magnetic layer to induce alternating Hall resistance. The rectification effect of alternating Hall resistance and alternating electric current produce second harmonic voltage which reflects the precession of magnetic moment under oscillating spin torque. For magnetic layer with perpendicular magnetic anisotropy (PMA) (e.g. Pt/Co/Pt), a unified approach and equation can be used to evaluate the SOT and determine the sign of SOT.


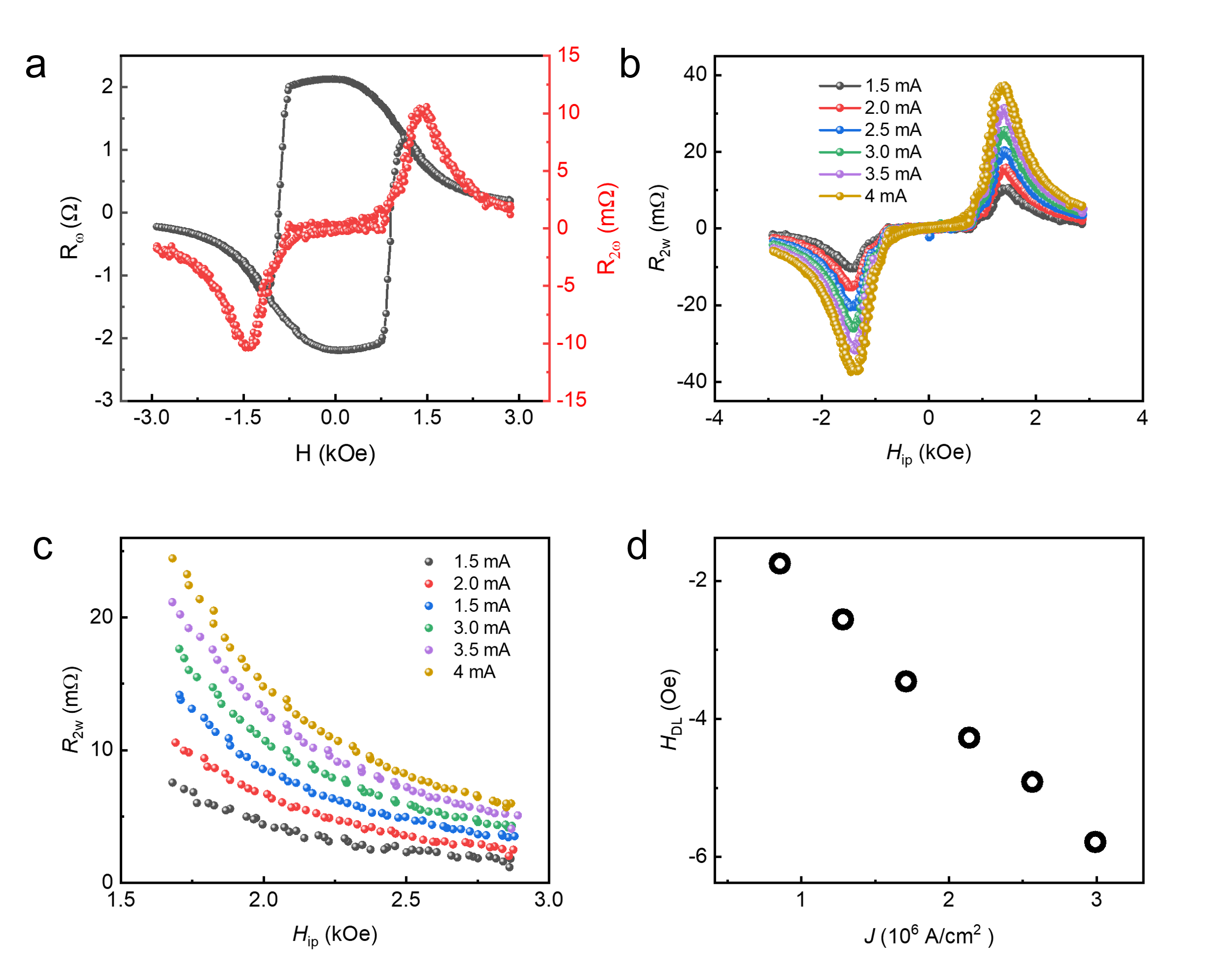


Figure S7. (a) The first and second harmonic *R*_1w_ and *R*_2w_ plotted against in-plane longitudinal magnetic field *H*_x_. (b-c) Current dependence of the *R*_2w_ in an in-plane longitudinal magnetic field *H*_x_. (d) The damping-like effective field *H*_DL_ as a function of current density for the Ni_50_Nb_50_(10)/Pt(1.5)/Co(0.7)/Pt(1.5 nm) sample.

For Pt/Co/Pt with PMA, we apply a large magnetic field 𝐻>𝐻_k_ (the magnetic anisotropy field) along x direction, so that magnetic moment is forced in-plane along x axis. For positive *H*_x_ along +x, 𝜑=0, while for negative *H*_x_ along –x, 𝜑=π. Accordingly, with negligible planar Hall effect in Pt/Co/Pt, the second harmonic resistance can be written as:

$R_{2w}=sgn(H_{x})\frac{1}{2}\frac{H_{DL}}{{|H}_{x}|{-H}_{k}}R_{A}$ (S1)


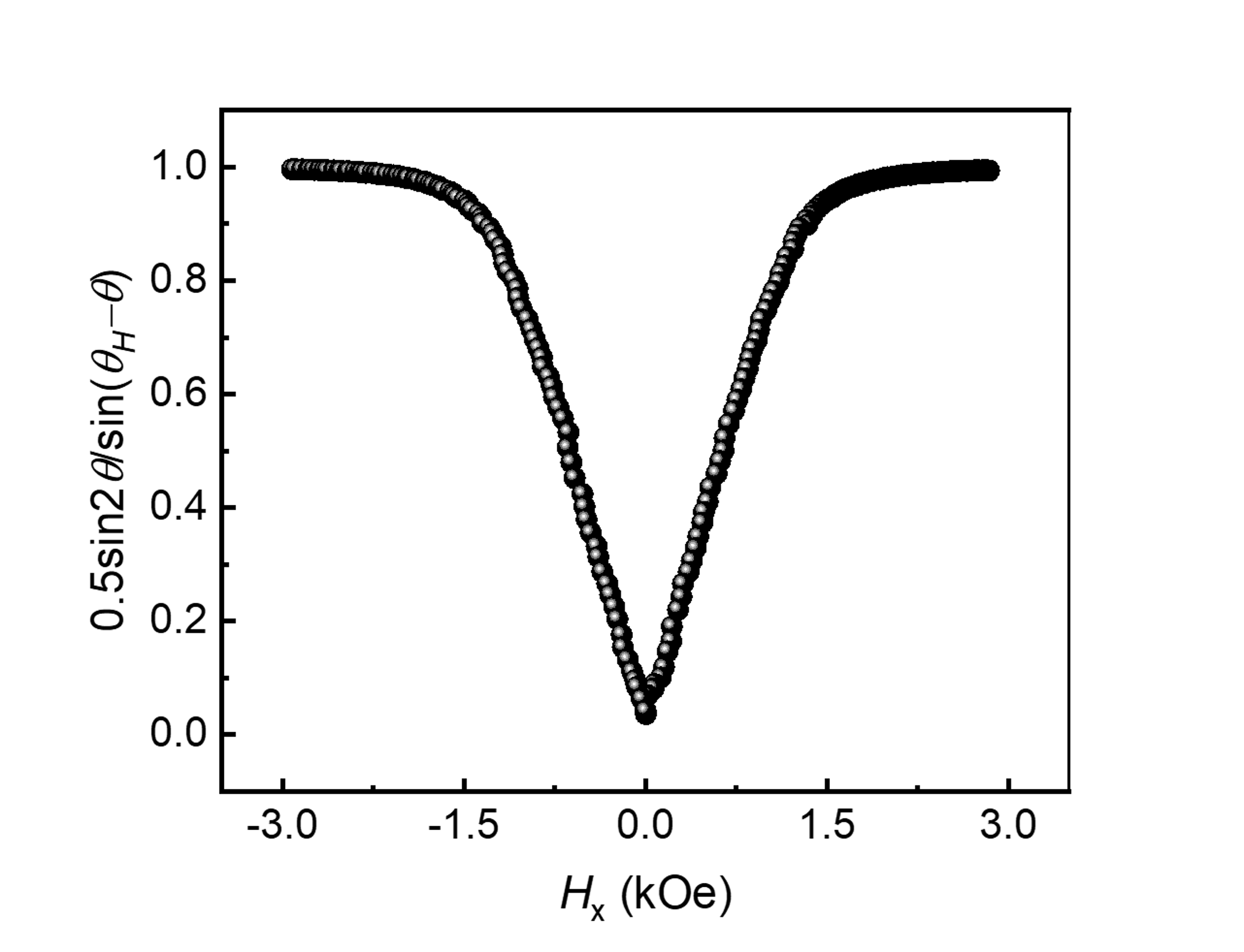
Accordingly, we utilized the anomalous Hall curves of each sample group to calculate the anisotropy field. Based on the equilibrium equation for the magnetic moment under a constant external magnetic field, sin2θ/sin(*θ*_H_-θ) =2*H*/*H*_k_ (where H, θ, *θ*_H_ and *H*_k_ represent the external magnetic field strength, the angle between the external magnetic field and the film normal, and the PMA field, respectively), if 0.5sin2θ/sin(*θ*_H_-θ) is expressed as a function of H, the reciprocal of its slope corresponds to the value of *H*_k_. Figure S8 illustrates the variation of 0.5sin2θ/sin(*θ*_H_-θ) with the in-plane magnetic field *H*_x_ for the control sample.

Figure S8. Variation of 0.5 sin2θ/sin(*θ*_H_-θ) for the Co layer magnetization with in-plane magnetic field *H*_x_.

Further measurement of *H*_DL_ at varied electric current can give the value of *H*_DL_/j shown in Fig. S6(d). The effective Hall conductivity characterizing the torque efficiency can be evaluated using the equation

$\sigma_{eff}=\frac{\hbar}{2e}\mu_{0}M_{s}t_{FM}\frac{H_{DL}}{j}$ (S2)


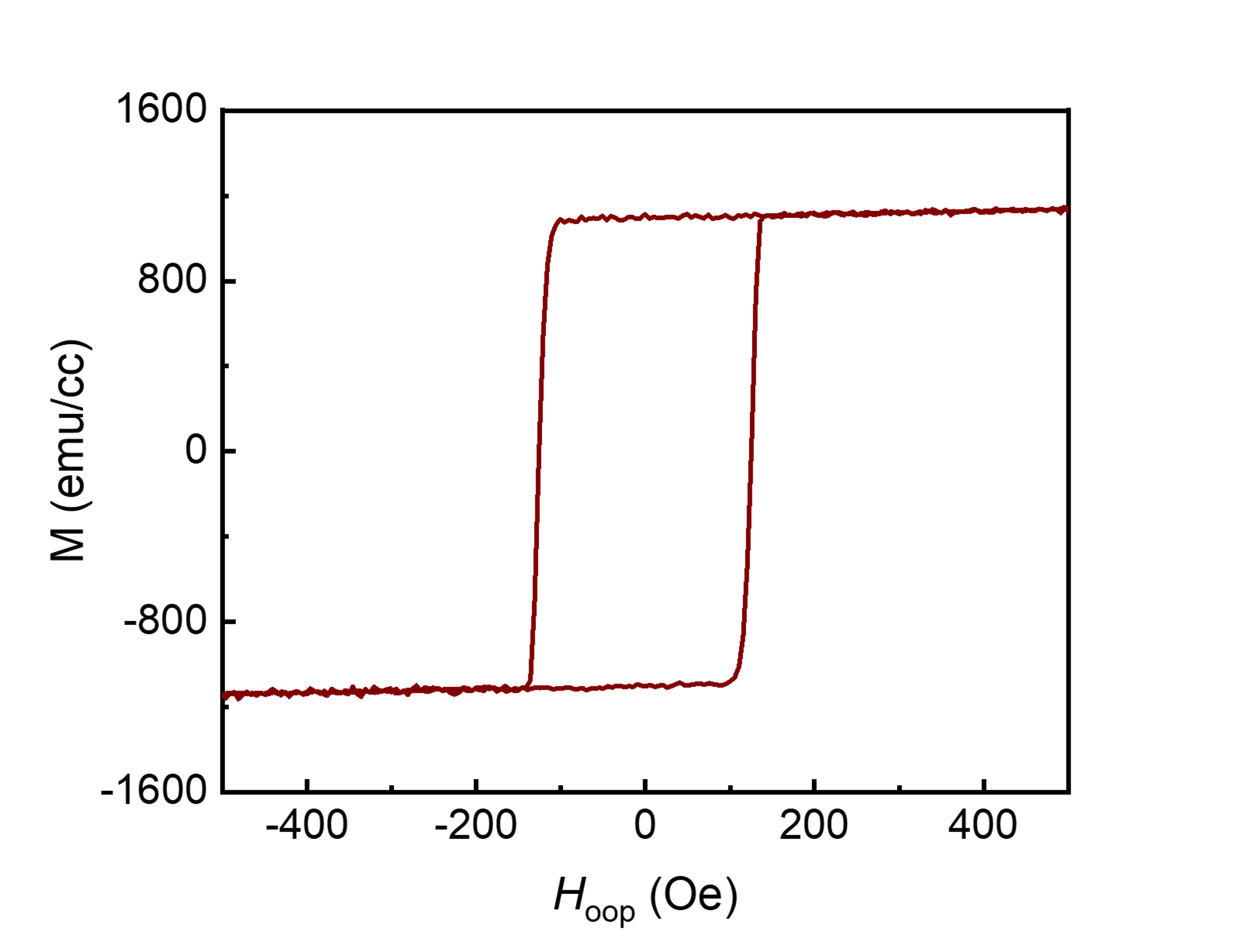
where 𝑒 is the electron charge, ℏ is the reduced Planck constant, 𝜇_0_ is the vacuum permeability *M*_s_(1100 emu/cc) is the magnetization of Pt/Co(0.7 nm)/Pt measured by SQUID as shown in Figure S9, and tFM is the thickness of Co.

Figure S9. M-H loops in a Pt(1.5)/Co(0.7)/Pt(1.5 nm) sample under an out-of-plane magnetic field.

# Note 6. SOT Efficiency for Different interlayer Thicknesses

We prepared samples of Ni_50_Nb_50_(10)/Pt(*t*_1_)/Co(0.7)/Pt(*t*_1_) and Ni_50_Nb_50_(10)/Ta(*t*_2_)/Pt(1.5) /Co(0.7)/Pt(1.5) with different interlayer thicknesses, where *t*_1_ = 1, 1.5, 2, 2.5 and 3 nm and *t*_2_ = 0.5, 1, 1.5, 2, 2.5 and 3 nm. We characterized its SOT efficiency using the second harmonic, and the damping-like effective field *H*_DL_ as a function of current density for the Ni_50_Nb_50_(10)/Pt(*t*_1_)/Co(0.7)/Pt(*t*_1_) in Figure S10(a) and Ni_50_Nb_50_(10)/Ta(*t*_2_)/Pt(1.5) /Co(0.7)/Pt(1.5) in Figure S10(b). The SOT efficiency *χ*_DL_=*H*_DL_/j as a function of interlayer thickness as shown in Figure S10(c). As the thickness of the Pt interlayer increases, its charge-to-spin conversion efficiency gradually decreases, whereas as the thickness of the Ta interlayer increases, its charge-to-spin conversion efficiency gradually increases. This further demonstrates that the spin current converted via the Pt interlayer from orbital currents that have not been fully converted in the Ni_50_Nb_50_ layer has the opposite sign to that converted in the Ni_50_Nb_50_ layer, whereas the spin current converted via the Ta interlayer has the same sign as that converted in the Ni_50_Nb_50_ layer. Therefore, as the thickness of the interlayer increases, the efficiency of the Pt interlayer decreases, while that of the Ta interlayer increases.

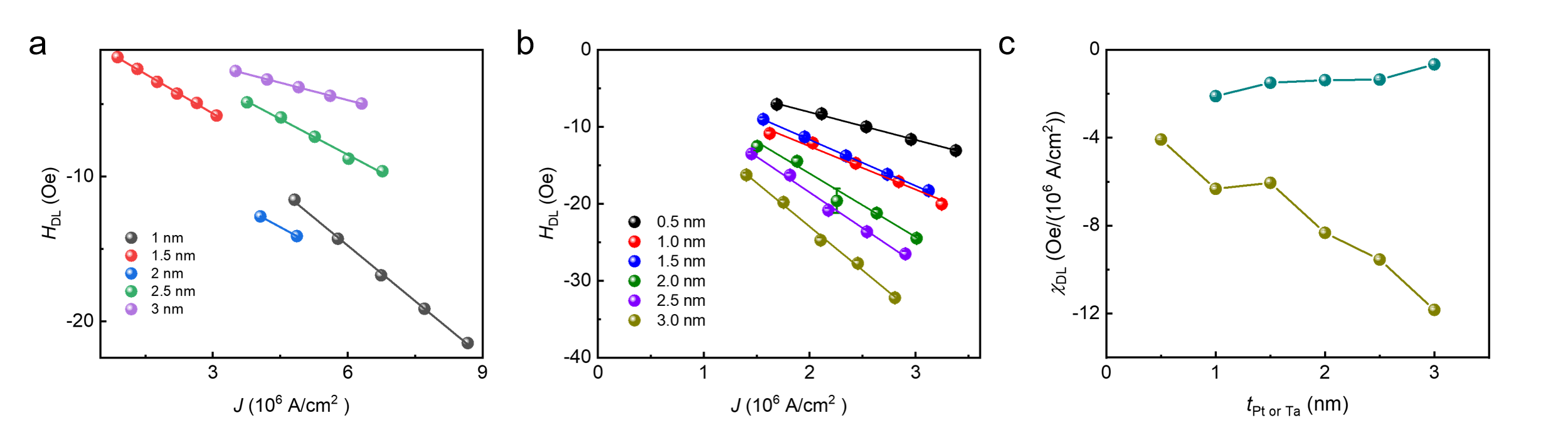


Figure S10 The damping-like effective field *H*_DL_ as a function of current density for the Ni_50_Nb_50_(10)/Pt(*t*_1_)/Co(0.7)/Pt(*t*_1_) (a) with different Pt thicknesses and Ni_50_Nb_50_(10)/Ta(*t*_2_)/Pt(1.5) /Co(0.7)/Pt(1.5) (b)with different Ta thicknesses. (c) The SOT efficiency *χ*_DL_ as a function of Pt or Ta thickness.

# Note 7. SOT Efficiency for Different Ni_50_Nb_50_ Layer Thicknesses

We prepared samples of Ni_50_Nb_50_(*t*)/Pt(1.5)/Co(0.7)/Pt(1.5) and Ni_50_Nb_50_(*t*)/Ta(1) /Pt(1.5) /Co(0.7)/Pt(1.5) with different Ni_50_Nb_50_ layer thicknesses, where t = 4, 6, 8, 10, 15, and 20 (nm). We characterized its SOT efficiency using the second harmonic technique, as shown in Figure S11. The damping-like effective field *H*_DL_ as a function of current density for the Ni_50_Nb_50_(t)/Pt(1.5)/Co(0.7)/Pt(1.5 nm) and Ni_50_Nb_50_(t)/Ta(1)/Pt(1.5)/Co(0.7) /Pt(1.5 nm). The SOT efficiency *χ*_DL_=*H*_DL_/j as a function of Ni_50_Nb_50_ thickness as shown in Figure S11(a). In both systems, the charge-spin conversion efficiency increases with increasing Ni_50_Nb_50_ thickness, and the charge-spin conversion efficiency in the Ta-inserted system is significantly higher than that in the system without Ta. This also indicates that the orbital current in the Ni_50_Nb_50_ is not fully converted; a portion of it is converted via the Pt or Ta at the interface. Because the signs of the SOC are opposite, the resulting spin current either increases (Ta) or decreases(Pt). However, the overall negative sign indicates that the spin polarization generated in Ni_50_Nb_50_ (negative) is greater than that converted in Pt (positive).

By fitting the thickness-dependent SOT efficiency (***𝜎***_eff_) using the equation:$\sigma_{eff}=\theta_{SH}^{eff}[1-sech(\frac{t}{\lambda_{L(S)}})]$, we extracted $\theta_{SH}^{eff}$ and the orbital (spin) diffusion length ($\lambda_{L(S)}$). The extracted *𝜆*_L_ is ∼6.7 ± 1.2 nm for the Ni_50_Nb_50_(t)/Pt(1.5)/Co(0.7)/Pt(1.5 nm) and 5.7 ± 0.4 nm for the Ni_50_Nb_50_(t)/Ta(1)/Pt(1.5)/Co(0.7)/Pt(1.5 nm), ∼4 times longer than the *𝜆*_S_ of ∼1.56 ± 0.12 nm for Ta. This is consistent with previous reports, where the orbital current penetrates a longer distance in solids compared to spin current.

Figure S11 (a) The SOT efficiency *χ*_DL_ as a function of NiNb thickness.

# Note 8. Current-driven magnetization switching

8.1 Magnetization switching measured at NiNb/CoTb

Fig. S12 shows the current-induced magnetization switching loop with an in-plane external field along the x direction.


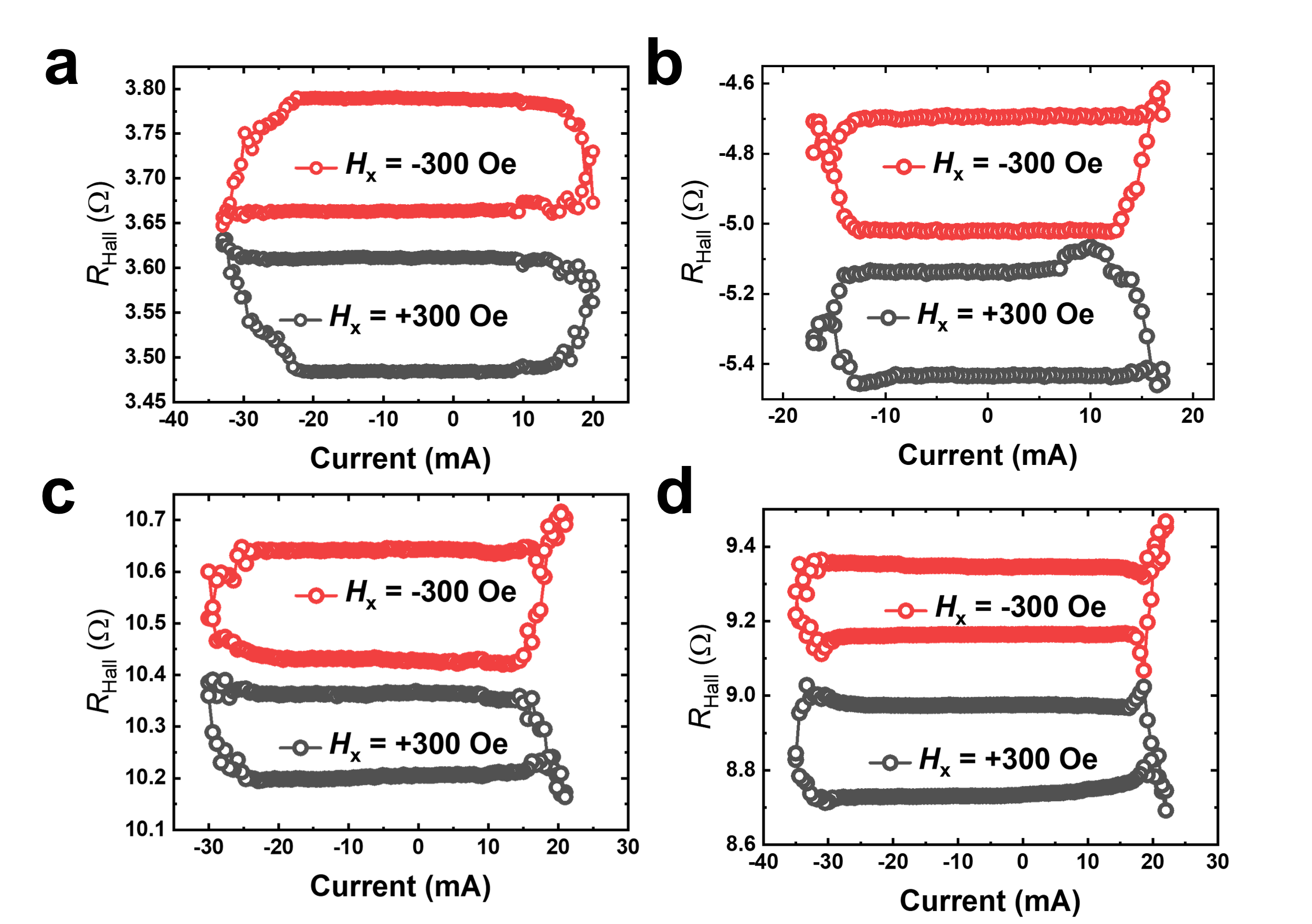


Fig. S12 Current-induced magnetization switching with the fixed in-plane magnetic field *H*_ext_ = +300 Oe or -300 Oe for the samples with different NiNb composition. (a) Ni_40_Nb_60_/CoTb/SiN, (b) Ni_45_Nb_55_/CoTb/SiN, (c) Ni_55_Nb_45_/CoTb/SiN and (d) Ni_60_Nb_40_/CoTb/SiN.

To evaluate the possible influence of current shunting, we have systematically summarized the resistivities of NiNb alloys with different compositions in Table 1. The results show that the resistivities of the NiNb alloys are lower than that of CoTb.


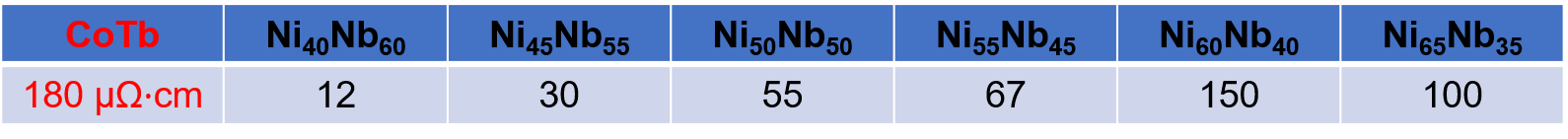
Table 1. Resistivities of CoTb and different composition NiNb alloys.

8.2 Magnetization switching measured at Ni_50_Nb_50_(*t*)/PMA.

To investigate SOT induced magnetization switching, we performed current-driven SOT switching measurements on samples with all different Ni_50_Nb_50_ layer thicknesses.

Figure S13 displays the magnetization switching at in-plane magnetic field with Ni_50_Nb_50_(*t*)/Pt(1.5)/Co(0.7)/Pt(1.5). To determine the critical switching current density for each Ni_50_Nb_50_ thickness, we summarize the current-driven magnetization switching under a positive auxiliary field (+200 Oe) as shown in the Figure S13 (a), the critical switching current density is shown in the Figure S13 (b), combining this with the SOT efficiency at different thicknesses (as shown in the Figure S10 (c)), we find that the critical switching current density is inversely proportional to the efficiency, as shown in the Figure S13 (c).

Figure S1
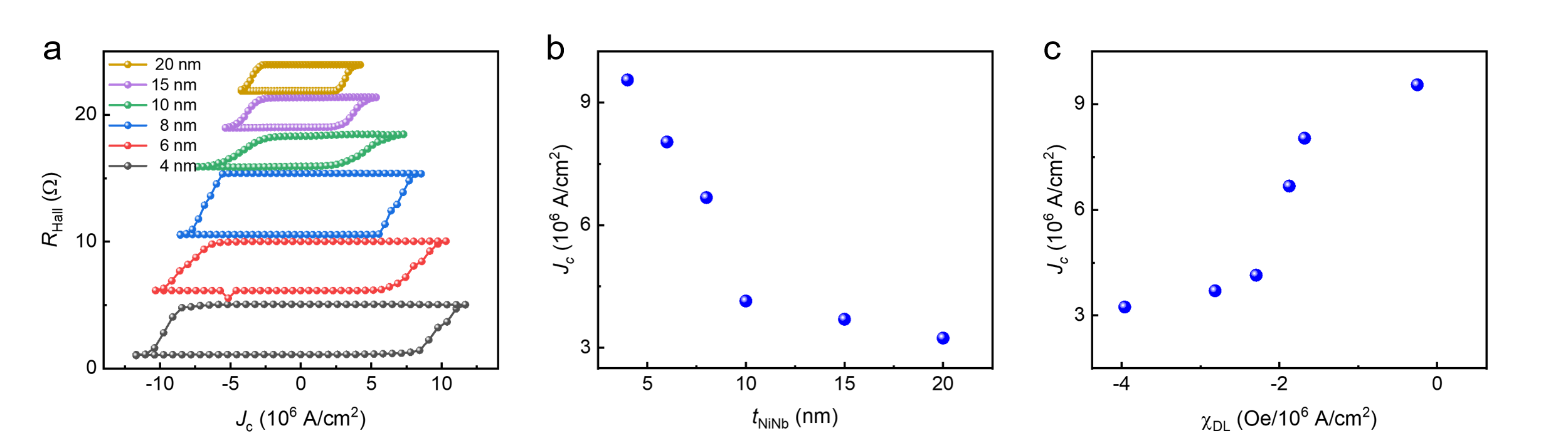
3 (a) Current-driven perpendicular magnetization switching in the Ni_50_Nb_50_(*t*)/Pt(1.5)/Co(0.7)/Pt(1.5) with different Ni_50_Nb_50_ thicknesses. (b) Dependence of the critical switching current density on Ni_50_Nb_50_ Thickness. (c) Dependence of the critical switching current density on the damping-like torque generated by different Ni_50_Nb_50_ thicknesses.

Following this, we have estimated the switching energy density using *u* = *J*_C_^2^*ρτ*, where *J*_C_, *ρ*, and *τ* is the critical switching current, resistivity and pulse width, respectively. As shown in Supporting Figure S14, the switching energy density of our NiNb/CoTb and NiNb/Pt/Co/Pt devices are estimated to be 0.25-1.22 μJ/cm^2^,
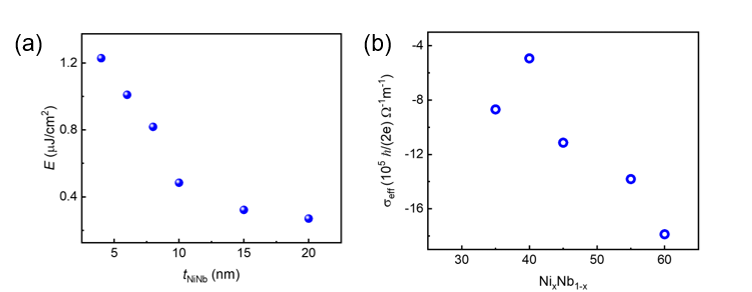
demonstrating the great potential of NiNb-based devices for low-power spintronic applications.

Figure S14 (a) Switching energy densities of NiNb/CoTb devices with different NiNb thicknesses.


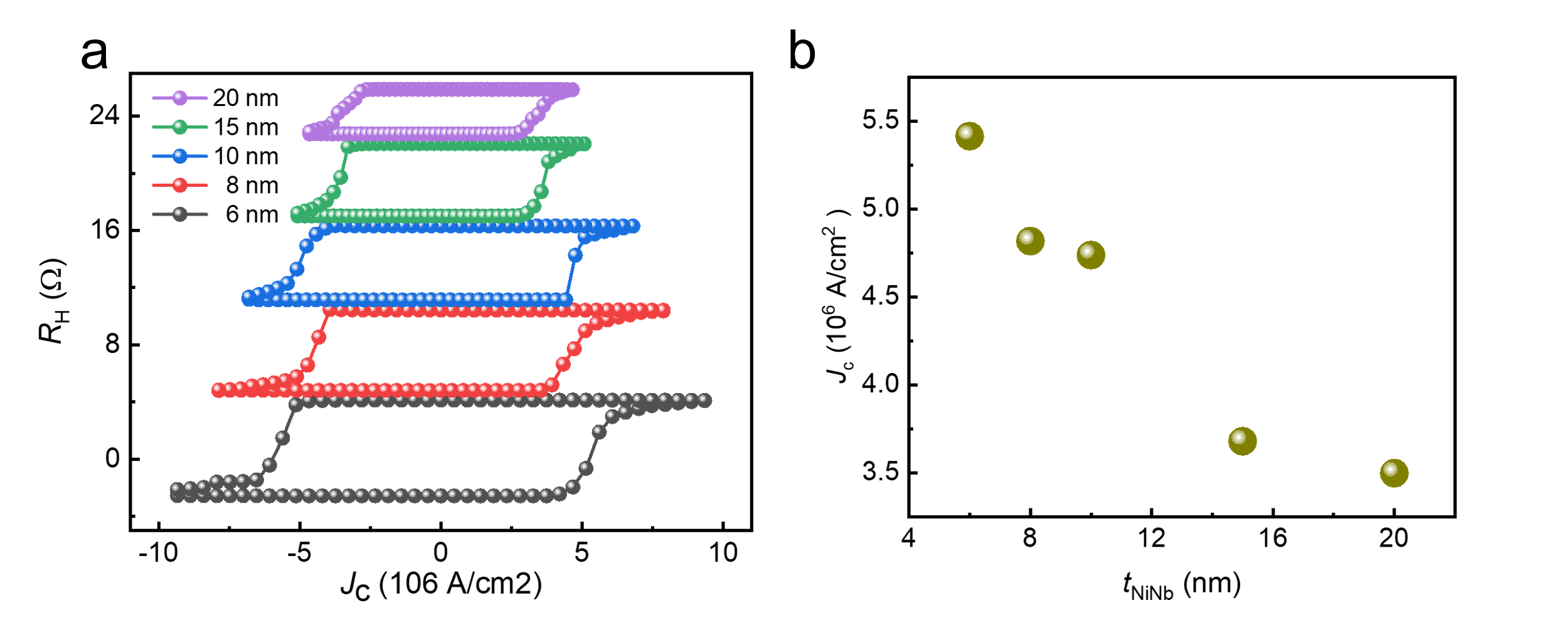


Figure S15 (a) Current-driven perpendicular magnetization switching in the Ni_50_Nb_50_(10)/Pt(*t*)/Co(0.7)/Pt(*t*) with the different thicknesses. (b) Dependence of the critical switching current density on Ta Thickness.

8.3 Magnetization switching measured at Ni_50_Nb_50_(8)/Pt(*t*)/Co(0.7)/Pt(*t* nm) and Ni_50_Nb_50_ (8)/Ta(t) /Pt(1.5)/Co(0.7)/Pt(1.5 nm).

Figure S16 displays the magnetization switching at in-plane magnetic field with Ni_50_Nb_50_(8)/Pt(t)/Co(0.7)/Pt(*t* nm), where t= 1, 1.5, 2, 2.5 and 3 nm.


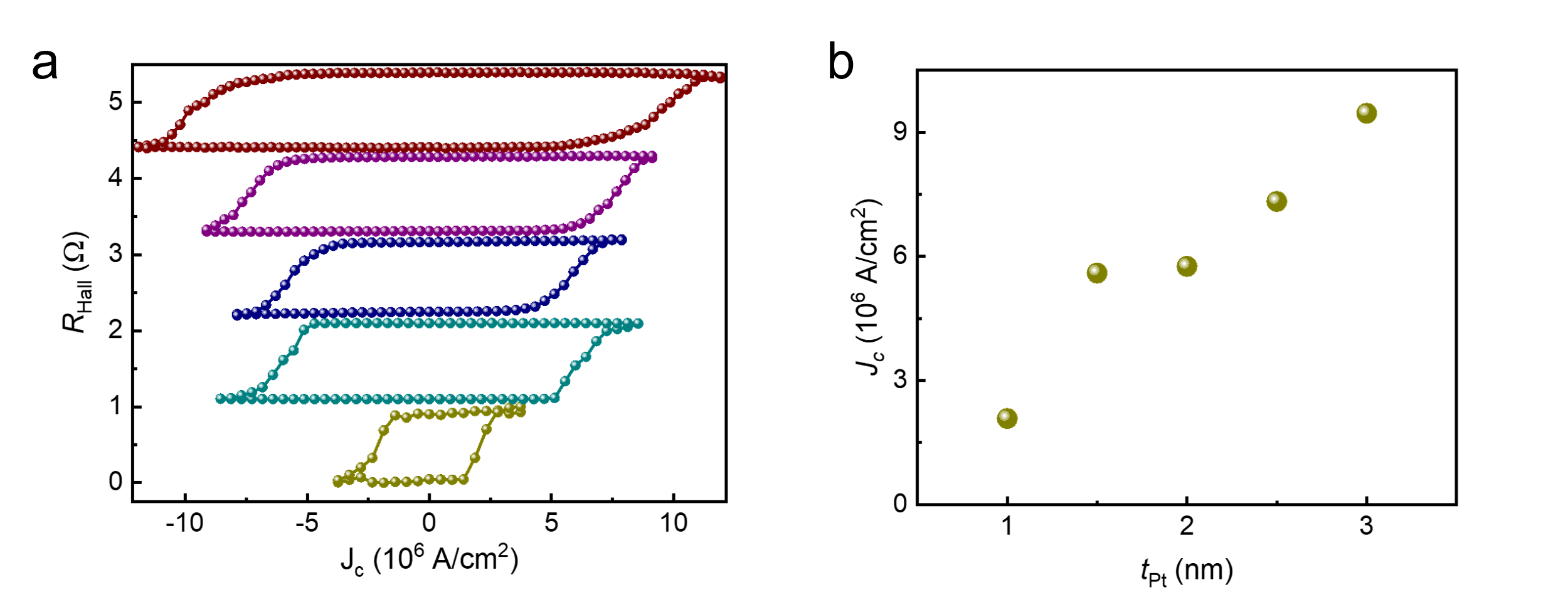


Figure S16 (a) Current-driven perpendicular magnetization switching in the Ni_50_Nb_50_(8)/Pt(*t*)/Co(0.7)/Pt(*t*) with the different Pt thicknesses. (b) Dependence of the critical switching current density on Pt tickness.


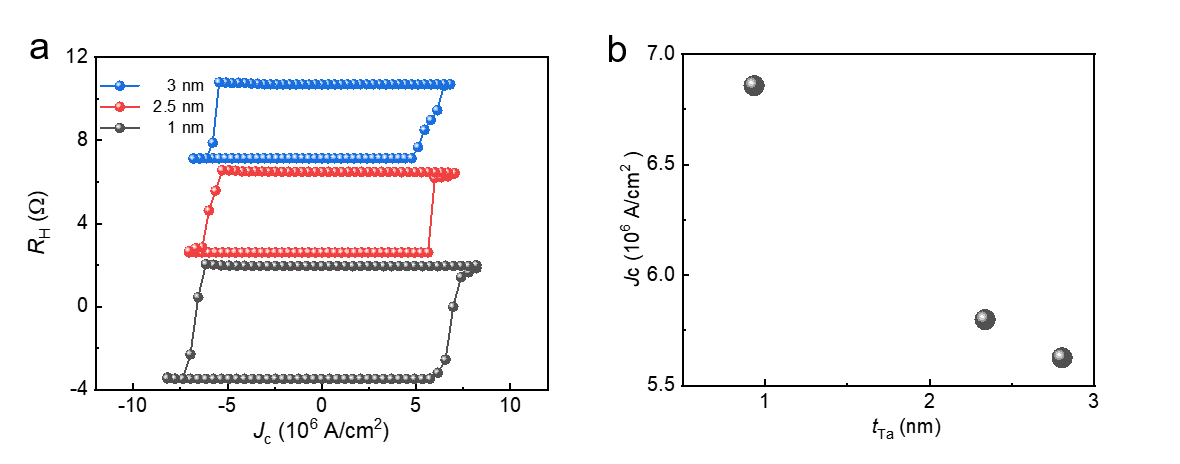
Figure S17 displays the magnetization switching at in-plane magnetic field with Ni_50_Nb_50_(8)/Ta(t) /Pt(1.5)/Co(0.7)/Pt(1.5 nm).

Figure S17 (a) Current-driven perpendicular magnetization switching in the Ni_50_Nb_50_(8)/Ta(*t*)/Pt(1.5)/Co(0.7)/Pt(1.5) with the different thicknesses. (b) Dependence of the critical switching current density on Ta Thickness.

8.4 Magnetization switching measured at Ni_50_Nb_50_(8)/Pt(1.5)/Co(0.7)/Pt(1.5 nm), Ni_50_Nb_50_(8)/CoTb(8)/SiN(3 nm), Nb(8)/ Pt(1.5)/Co(0.7)/Pt(1.5 nm) and Nb(8)/CoTb(8) /SiN(3 nm).

We grew Pt(1.5)/Co(0.7)/Pt(1.5 nm) and CoTb/capping perpendicular magnetic anisotropy layers on Ni_50_Nb_50_ and Nb, respectively. All four samples exhibit good perpendicular magnetic anisotropy, as demonstrated by the field-driven anomalous Hall loops as shown in the Figure S18. Furthermore, for the PMA-Layer (Pt(1.5)/Co(0.7)/Pt(1.5 nm)) vertical with strong spin-orbit coupling, current-driven switching of the perpendicular magnetization can be achieved with both Ni_50_Nb_50_ and Nb; however, for the PMA-CoTb samples, current-driven switching of the perpendicular magnetization is only achievable in the Ni_50_Nb_50_ alloy samples exhibiting strong spin Hall and orbital Hall effects. Pure Nb samples are unable to achieve perpendicular magnetization reversal in CoTb, thereby demonstrating that in our system, the SOC of rare earth elements is insufficient to facilitate the orbital-to-spin conversion in Nb. And as shown in Figure S19, the critical switching current density in the Pt/Co/Pt sample is slightly higher than that in the CoTb sample. These results further confirm the reliability of our characterization of the NiNb/CoTb samples, and the slightly lower critical switching current density in the CoTb-based sample can be attributed to the negative exchange interaction in the ferrimagnetic layer.


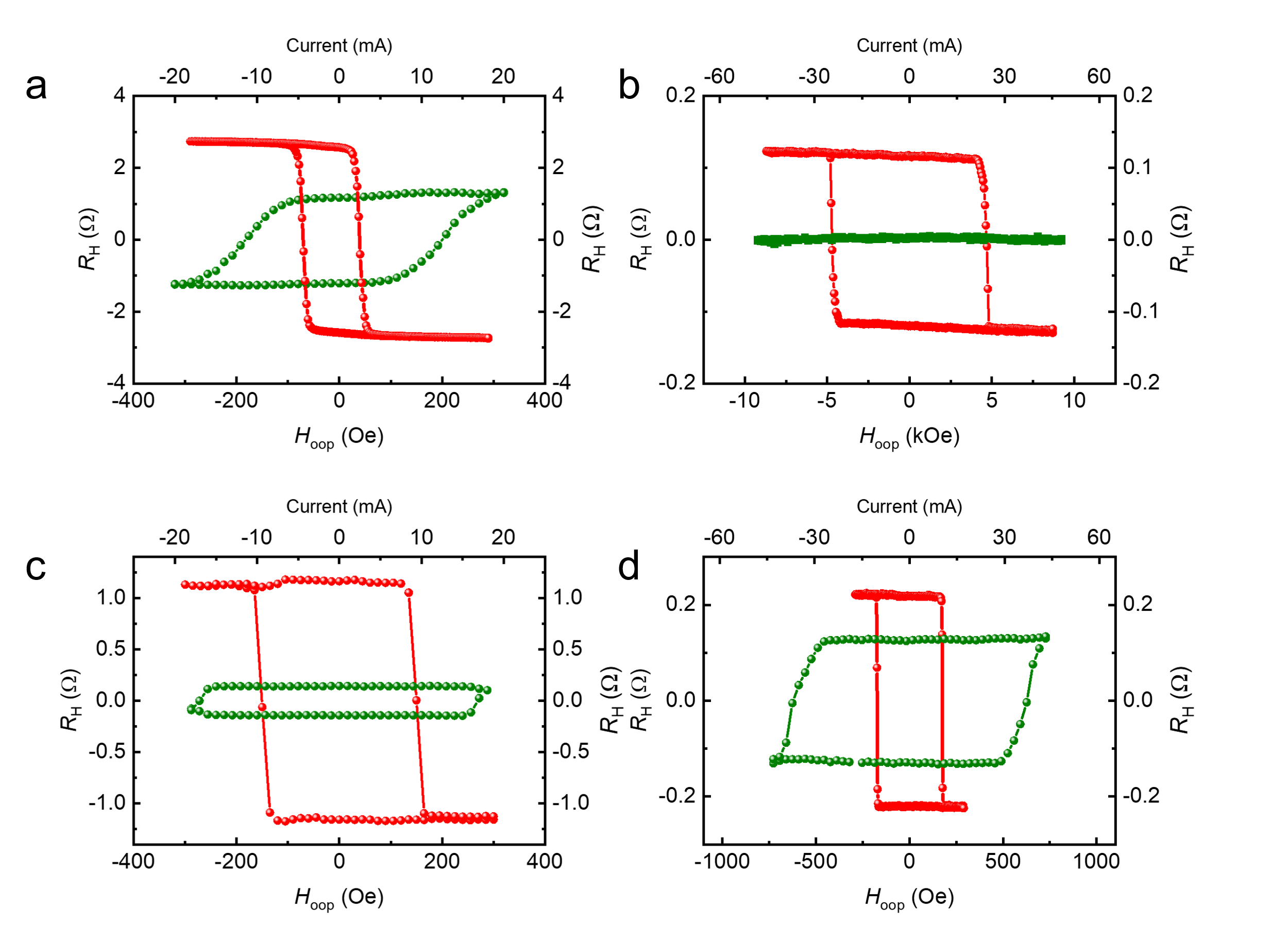
Figure S18 The magnetization switching at in-plane magnetic field with (a) Ni_50_Nb_50_(10)/Pt(1.5)/Co(0.7)/Pt(1.5), (b) Nb(8)/Pt(1.5)/Co(0.7)/Pt(1.5), (c) Ni_50_Nb_50_(10)/CoTb(8)/SiN and (d) Nb(8)/CoTb(8)/SiN. The red lines represent anomalous Hall loops in which the magnetic field drives the switching of magnetization, and the green lines represent current-driven magnetization reversal loops, with an in-plane auxiliary field of −200 Oe parallel to the current direction.


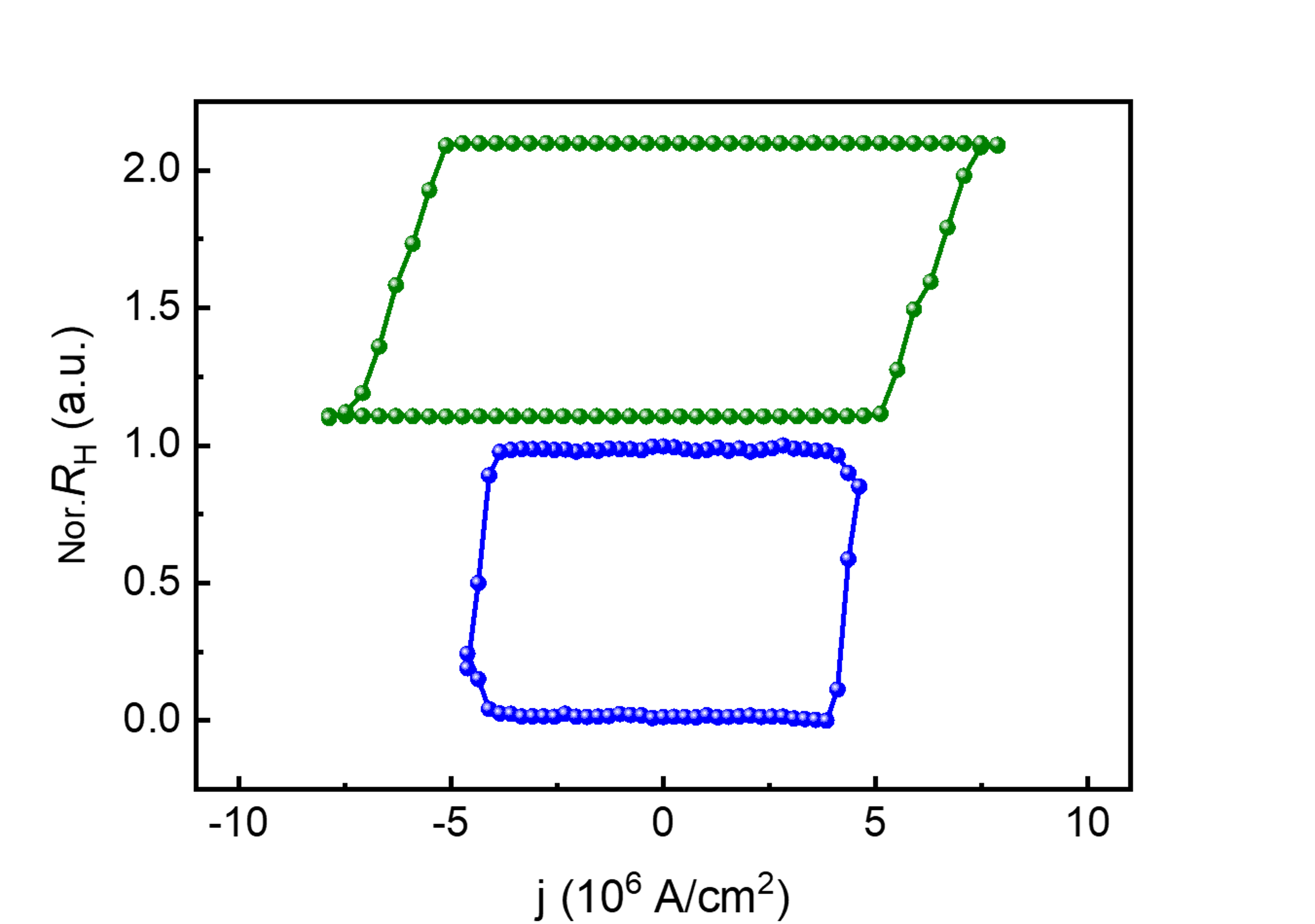
Figure S19. Current-driven perpendicular magnetization switching in NiNb(10)/ CoTb(8)/SiN(3 nm) (blue) and NiNb(10)/Pt(1.5)/Co(0.7)/Pt(1.5 nm) (green) samples.

# Note 9. SOT-Driven perpendicular Magnetization Switching Ratio

Furthermore, we summarized the switching ratio for all samples as all different Ni_50_Nb_50_ layer thicknesses. The maximum Δ*R*_H_ for samples different Ni_50_Nb_50_ layer thicknesses in Field-driven magnetization switching are defined as Δ*R*_max_. The ratio of Δ*R*_H_ to Δ*R*_max_ for samples with different Ni_50_Nb_50_ layer thicknesses in current-driven magnetization switching is switching ratio and it can be expressed as the following equation:

$Switching ratio = \frac{\Delta R_{H}}{\Delta R_{Max}}$ (S3)

It can be observed that the switching ratio of Pt/Co/Pt heterojunctions grown on Ni_50_Nb_50_ layers of different thicknesses varies slightly, however, the magnitude of this variation is very small, its switching ratio is generally greater than 80% as shown in Figure S20, which is significantly higher than that of the vertical layer consisting of CoTb.

The multidomain switching behavior of CoTb can also be observed from our MOKE data (Figure S21). Therefore, the partial switching in the Ni_50_Nb_50_/CoTb devices does not necessarily indicate weak torque generation. To further verify the switching capability of Ni_50_Nb_50_-based devices, we measured the current-induced magnetization switching in Ni_50_Nb_50_/Pt/Co/Pt control samples.

Figure S20 switching ratio at in-plane magnetic field *H*_x_ = - 200 Oe with (a) Ni_50_Nb_50_ (*t*)/Ta(1)/Pt(1.5)/Co(0.7)/Pt(1.5 nm), (b) Ni_50_Nb_50_(10)/Ta(*t*)/Pt(1.5)/Co(0.7)/Pt(1.5).


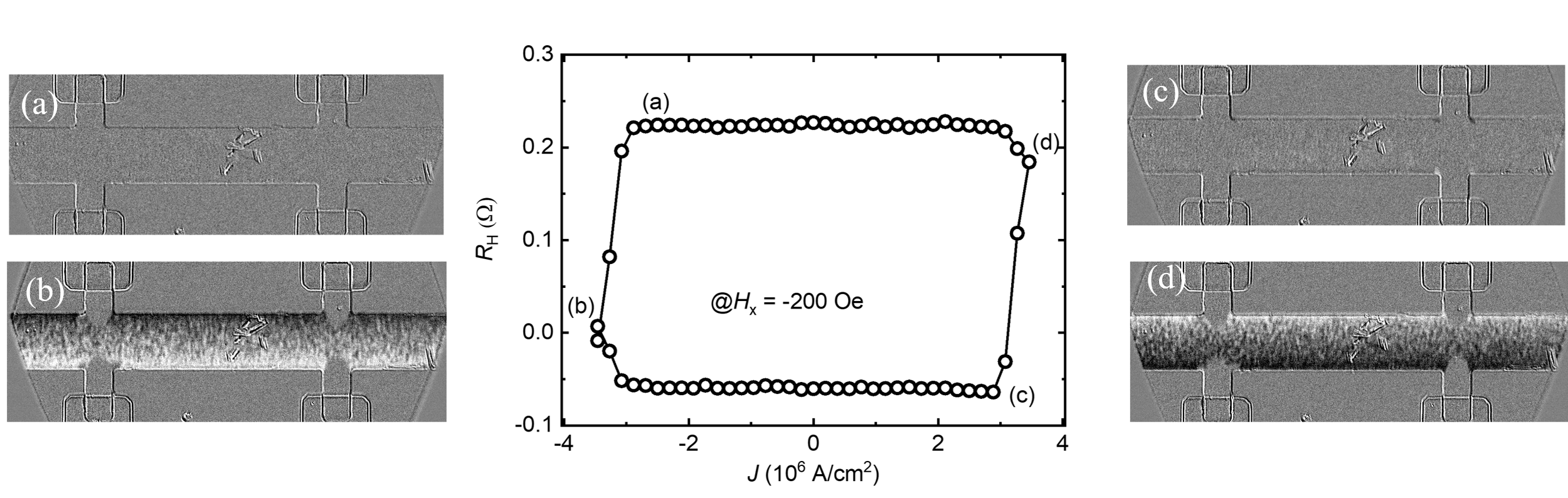


Figure S21 Current-Driven Magnetization switching in Ni_50_Nb_50_/CoTb Samples and The MOKE images.

# Note 10. Comparison of Samples from Traditional Heavy Metal Systems

For samples exhibiting in-plane magnetic anisotropy, we grew Pt(8)/Py(8)/SiN(3 nm) and Ta(8)/Py(8)/SiN(3 nm) samples. The spin-torque efficiencies of the Pt and Ta reference samples characterized by spin-torque ferromagnetic resonance are estimated to be 0.12 and -0.145, respectively, which are in good agreement with previously reported values. More importantly, as shown in the Figure S22, their spin-torque ferromagnetic resonance signals indicate that their charge-to-spin conversion efficiency is significantly lower than that of Ni_50_Nb_50_/Py/SiN.

For samples with perpendicular magnetic anisotropy, we grew Ta(8)/Pt(1.5)/Co(0.7)/Pt(1.5) samples. The current-driven perpendicular magnetization reversal under an auxiliary field is shown in the Figure S23 indicating that the critical reversal current density is much higher than that of Ni_50_Nb_50_/Pt/Co/Pt.
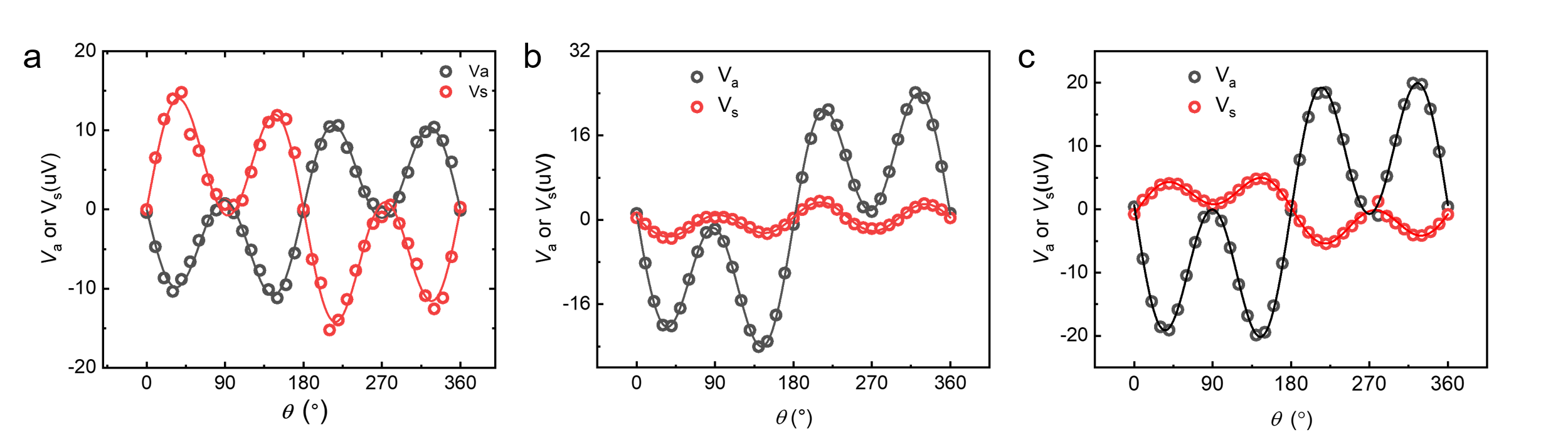


Figure S22 *V*_a_ and *V*_s_ voltage signals as a function of *θ*. (a) Ni_50_Nb_50_(10)/Py(8)/SiN(3 nm). (b) Pt(8)/Py(8)/SiN(3 nm). (c) Ta(8)/Py(8)/SiN(3 nm)


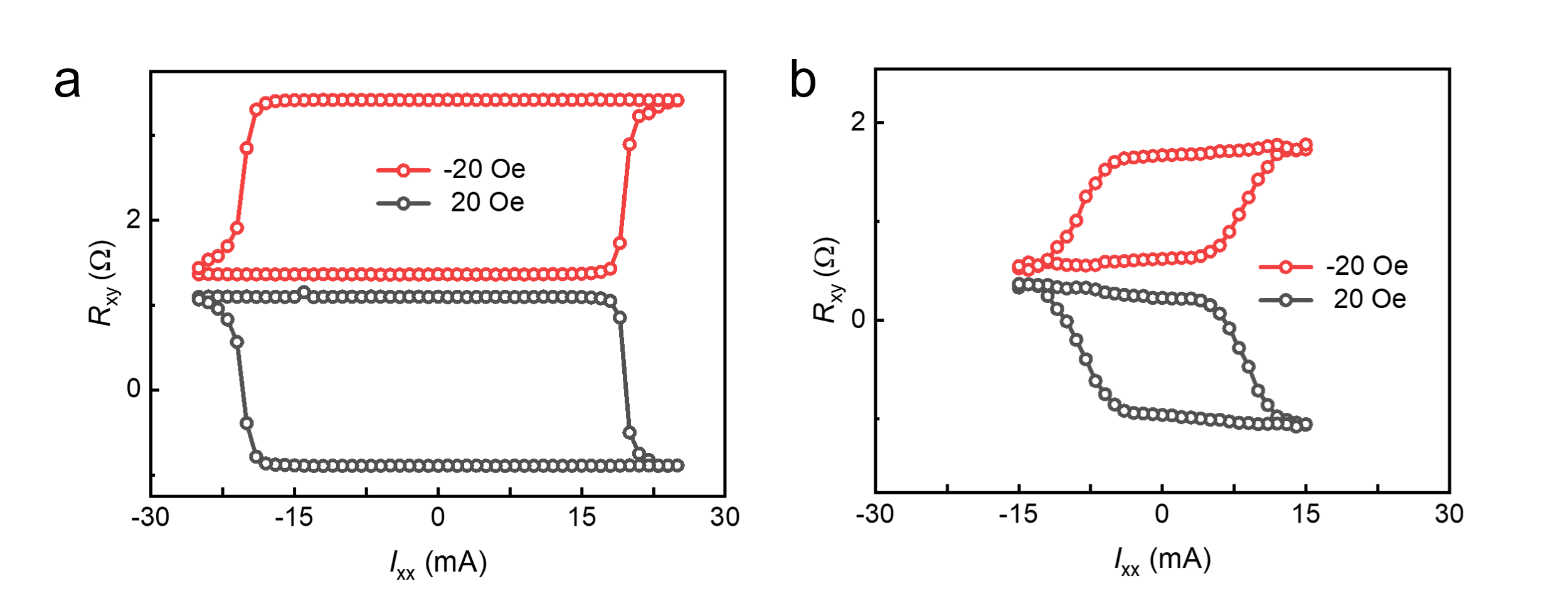


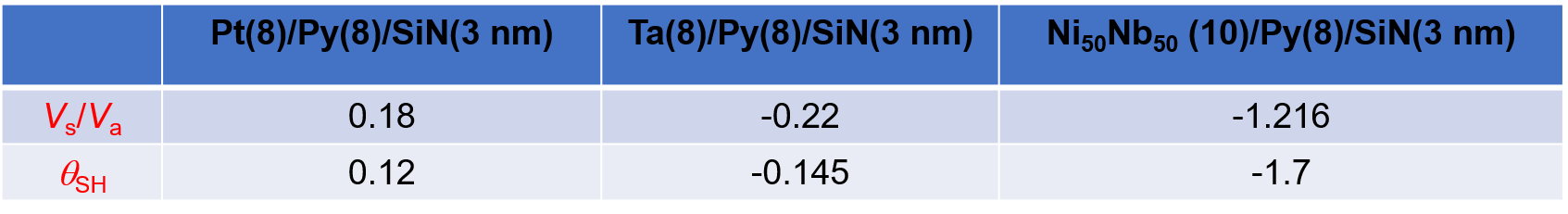
Figure S23 Current-Driven Magnetization switching. (a) Ta(8)/Pt(1.5)/Co(0.7)/Pt(1.5). (b) Ni_50_Nb_50_ (8)/Pt(1.5)/Co(0.7)/Pt(1.5).

Table2. Summary of *V*_s_*/V*_a_ and *θ*_SH_ for NiNb(8)/Py(8)/SiN(3 nm), Pt(10)/Py(8)/SiN(3 nm) and Ta(8)/Py(8)/SiN(3 nm).

# Note 11. Temperature-dependent charge-spin conversion efficiency

Fig. S24 shows that at different temperatures, the *I*_rf_ was applied to the device with a frequency of 6 GHz and power amplitudes from 10 dBm to 35 dBm (1 mW = 10^0.1*1 dBm^).

Fig. S25 shows the resonance field dependence of the microwave frequency and the linewidth dependence of the microwave frequency. According to the Kittel equation for in-plane ferromagnetic films:

$f=\frac{\gamma}{2\pi}\sqrt{\left( {4\pi M}_{eff}+H \right)H}$ (S4)

in which 4π*M*_eff_ is effective magnetization, *H* is magnetic field. The ST-FMR linewidth as a function of microwave frequency. The Gilbert damping can be obtained by the fitting formula:

$\Delta H=\Delta H_{0}+2\alpha\omega/\gamma$ (S5)

where Δ*H* is ST-FMR linewidth, Δ*H*_0_ is the inhomogeneous linewidth, which is independent of microwave frequency, ω = 2πf is microwave angle frequency, γ is the gyromagnetic ratio, and α is the dimensionless Gilbert damping constant.

Fig. S26(a) shows that the Gilbert damping constant α and effective demagnetization field 4π*M*_eff_ remain essentially constant over the measured temperature range. Fig. S26 (b) shows the spin-orbit torque efficiency at different temperatures, and the inset shows the dependence of the resistivity of the spin-source layer Ni_45_Nb_55_ on temperature. We find that at low temperatures, its resistivity does not change by a large amount (13.4%) compared to room temperature (300 K), but its SOT efficiency increases by a factor of 237%, thus suggesting that the orbitals and spins synergistically work together to produce a greater charge-spin conversion efficiency.


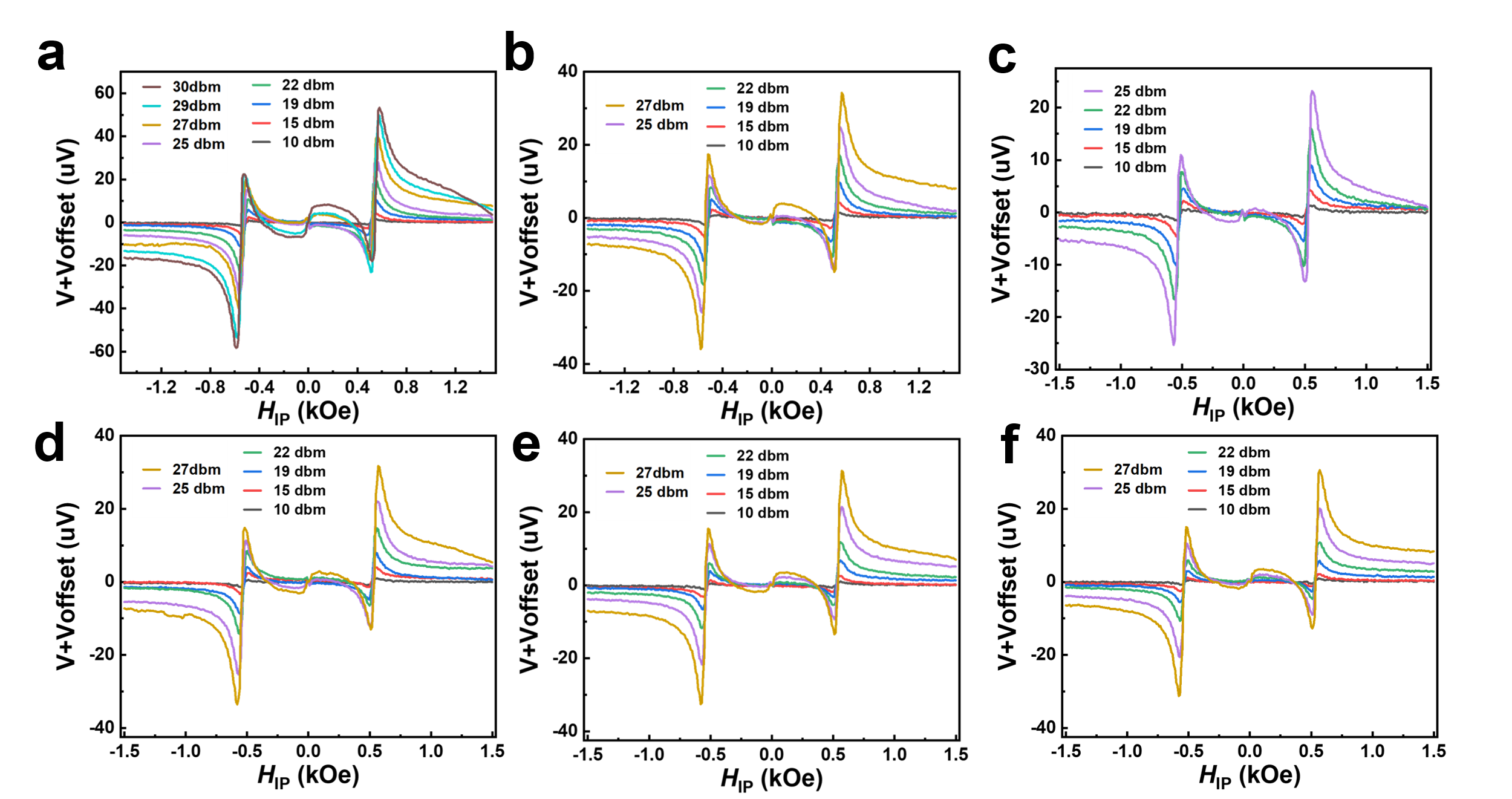

Fig. S24. Spin torque ferromagnetic resonance signals of Ni45Nb55/Py at low temperatures and different microwave powers. (a) 5 K, (b) 50 K, (c) 100 K, (d) 150 K, (e) 200 K, and (f) 250 K.


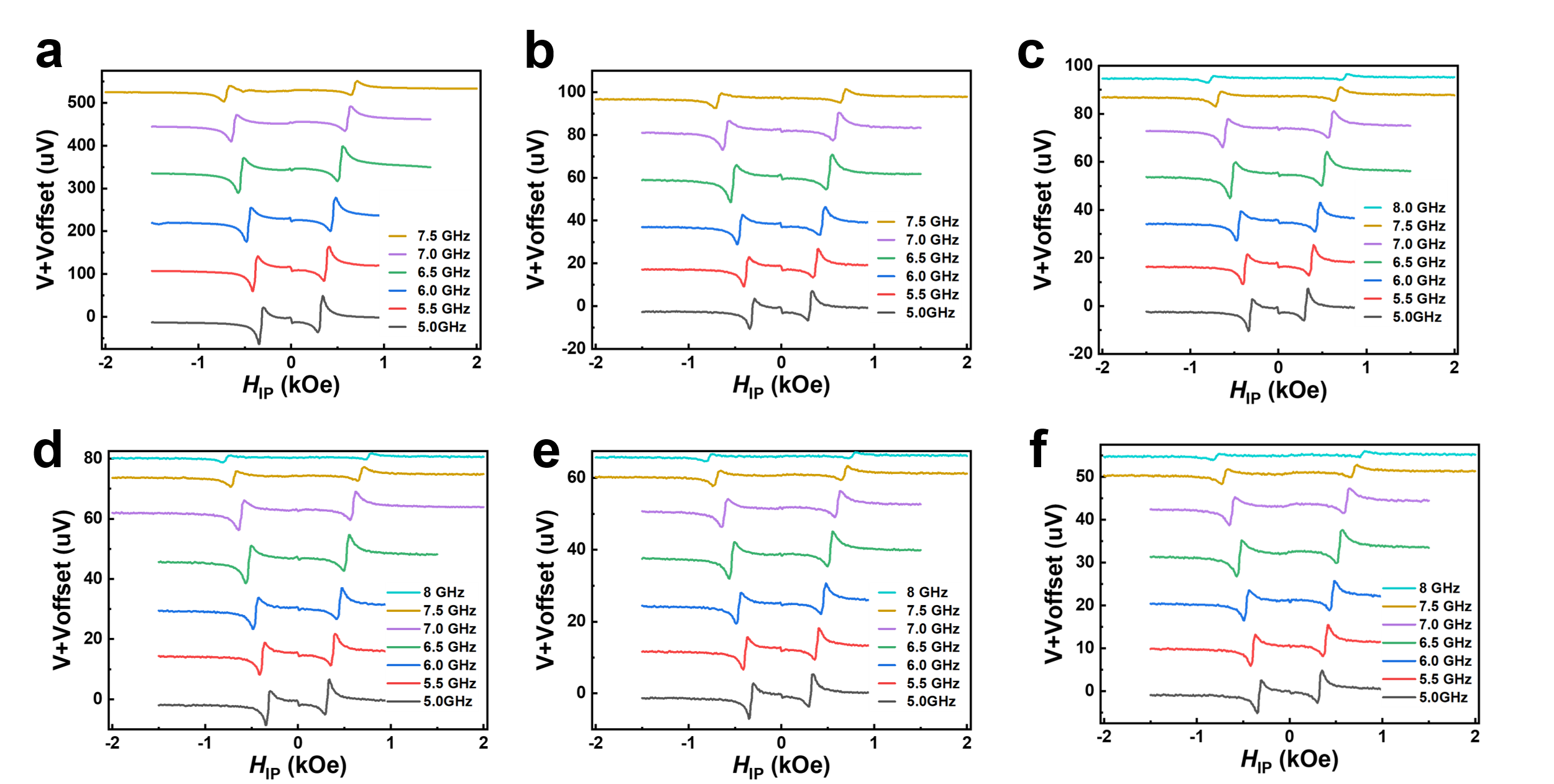
 Fig. S25. Spin torque ferromagnetic resonance signals of Ni45Nb55/Py at low temperatures and different microwave frequencies. (a) 5 K, (b) 50 K, (c) 100 K, (d) 150 K, (e) 200 K, and (f) 250 K.


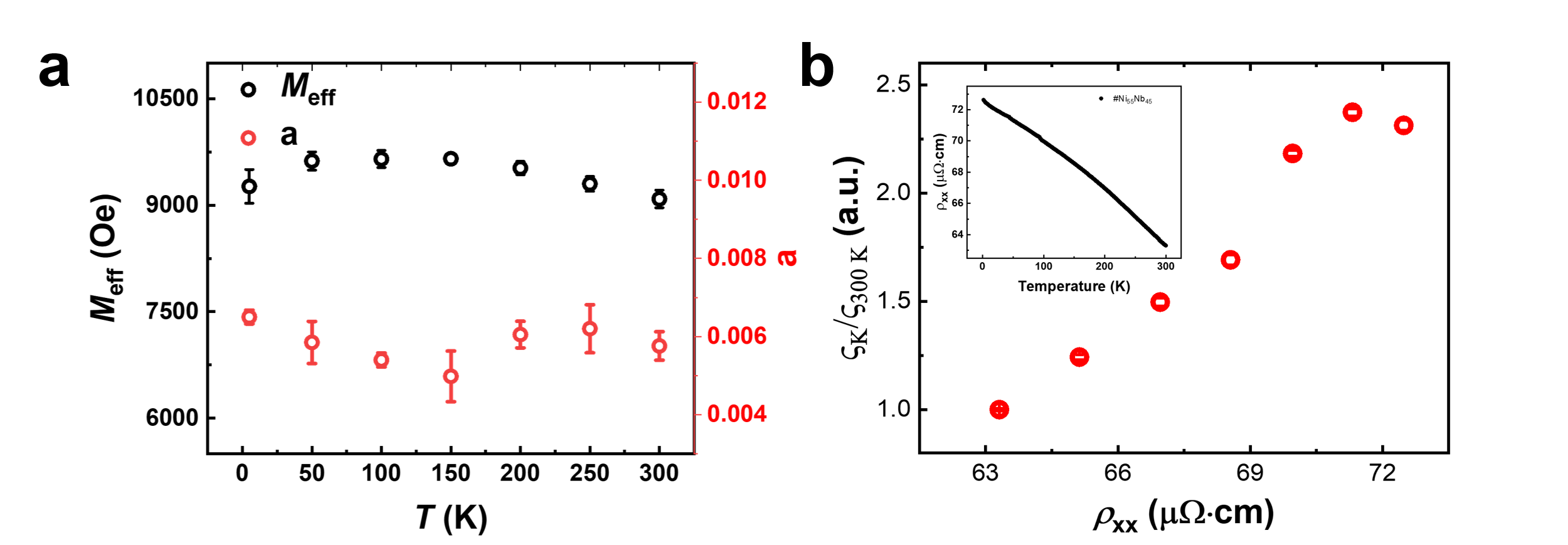


Fig. S26 (a) Dependence of effective magnetization strength 4π*M*_eff_ and Gilbert damping constant α with temperature d. (b) Temperature dependence of spin efficiency. The inset shows the R-T of Ni_45_Nb_55_.

# Note12. Theoretically calculated orbital hybridization of Ni and Nb

Fig. S27 shows the distribution of Density of states for Ni and Nb in NiNb alloys with different Nb contents. We observe that when Ni and Nb are present in equal amounts, the orbital hybridization between the two elements is strongest.


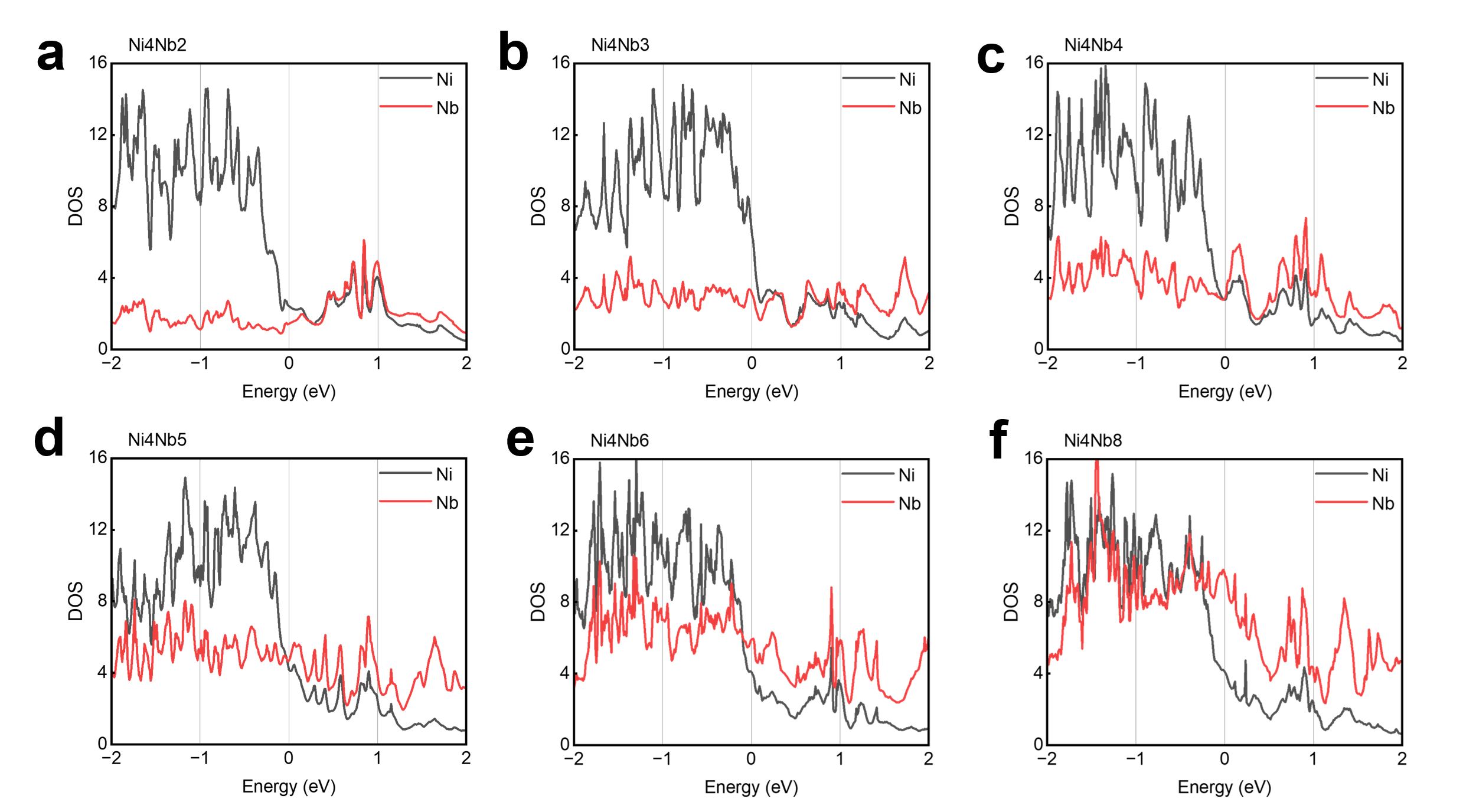
 Fig. S27. Electronic density of states distribution of Ni and Nb at different compositions. (a) Ni_4_Nb_2_, (b) Ni_4_Nb_3_, (c) Ni_4_Nb_4_, (d) Ni_4_Nb_5_, (e) Ni_4_Nb_6_ and (f) Ni_4_Nb_8_.
